# VarLOCK: sequencing-independent, rapid detection of SARS-CoV-2 variants of concern for point-of-care testing, qPCR pipelines and national wastewater surveillance

**DOI:** 10.1038/s41598-023-47289-0

**Published:** 2023-11-27

**Authors:** Xinsheng Nan, Patrick Hardinge, Sven Hoehn, Shrinivas Nivrutti Dighe, John Ukeri, Darius F. Pease, Joshua Griffin, Jessica I. Warrington, Zack Saud, Emma Hottinger, Gordon Webster, Davey Jones, Peter Kille, Andrew Weightman, Richard Stanton, Oliver K. Castell, James A. H. Murray, Tomasz P. Jurkowski

**Affiliations:** 1 Cardiff School of Biosciences, Cardiff University, Sir Martin Evans Building, Museum Avenue, Cardiff, CF10 3AX UK; 2 Cardiff School of Pharmacy and Pharmaceutical Sciences, Cardiff University, Redwood Building, King Edward VII Avenue, Cardiff, CF10 3NB UK; 3 COVID-19 Screening Service, Cardiff University, Sir Martin Evans Building, Museum Avenue, Cardiff, CF10 3AX UK; 4 School of Natural Sciences, Bangor University, Bangor, Gwynedd LL57 2UW UK; 5 Infection and Immunity, School of Medicine, Cardiff University, Heath Park, Cardiff, CF14 4XN UK; 6 Present Address: Biodexa Pharmaceuticals (Wales) Ltd, 1 Caspian Point, Caspian Way, Cardiff CF10 4DQ UK

**Keywords:** Nucleic acids, Viral infection, Biochemical assays

## Abstract

The COVID-19 pandemic demonstrated the need for rapid molecular diagnostics. Vaccination programs can provide protection and facilitate the opening of society, but newly emergent and existing viral variants capable of evading the immune system endanger their efficacy. Effective surveillance for Variants of Concern (VOC) is therefore important. Rapid and specific molecular diagnostics can provide speed and coverage advantages compared to genomic sequencing alone, benefitting the public health response and facilitating VOC containment. Here we expand the recently developed SARS-CoV-2 CRISPR-Cas detection technology (SHERLOCK) to provide rapid and sensitive discrimination of SARS-CoV-2 VOCs that can be used at point of care, implemented in the pipelines of small or large testing facilities, and even determine the proportion of VOCs in pooled population-level wastewater samples. This technology complements sequencing efforts to allow facile and rapid identification of individuals infected with VOCs to help break infection chains. We show the optimisation of our VarLOCK assays (Variant-specific SHERLOCK) for multiple specific mutations in the S gene of SARS-CoV-2 and validation with samples from the Cardiff University Testing Service. We also show the applicability of VarLOCK to national wastewater surveillance of SARS-CoV-2 variants and the rapid adaptability of the technique for new and emerging VOCs.

## Introduction

Genetic variations in the SARS-CoV-2 genome emerge during viral replication. Whilst mutations are often inconsequential, several mutations emerged during the pandemic conferring phenotypic advantages to the virus and posing additional challenges for national and global COVID-19 responses. To date the World Health Organisation (WHO) has assigned five Variants of Concern (VOC) labelled: Alpha—B.1.1.7, Beta—B.1.351, Gamma—P.1, Delta—B.1.617.2 and most recently Omicron—B.1.1.529^1^. These variants display increased transmissibility (Alpha< Delta< Omicron), increased risk of severe illness and hospitalisation (Alpha) and raised concerns about immune response evasion (Beta, Delta, Omicron), posing increased potential risks of re-infection and vaccine escape combined with increased transmissibility. Such variants pose a risk of immune escape and their surveillance have been a longstanding point of concern.

Genomic sequencing has been extremely successful in identifying new variants, in the tracking of viral lineages to understand viral introduction events and transmission^2^, as well as monitoring virus evolution during the pandemic in near real-time. Genomic sequencing remains the gold-standard for tracking viral mutations and identifying emerging Variants of Concern. Once specific mutations of VOCs are known, which may be identified anywhere in the world, sequencing becomes a powerful indicator of local prevalence, but it is also a lagging indicator due to the high resource requirements and potential delays in sequencing. In practice, this currently means results take several days following a PCR positive SARS-CoV-2 test and only a sub-set of positive patient samples are typically sequenced, corresponding to typically circa 10% of positive identified cases in the UK during the pandemic, but varies geographically. This delay and sub-sampling impacts the speed and efficacy of the public health response as it is highly likely that once known VOCs are identified through genomic sequencing, multiple other undetected cases in the community will already be present.

There is therefore a requirement for combining genetic virus surveillance by sequencing with sequence-specific molecular diagnostics for rapid discrimination of variants in either laboratory or point of care testing scenarios, as well as in wastewater samples which provide virus and variant surveillance at a local and national level. This has the potential to boost the speed and efficacy of the public health response to new and emerging VOCs, here illustrated with the example of SARS-CoV-2. Such sequence-specific tests, including nucleic acid amplification and CRISPR-Cas cleavage techniques, rely on sequence complementarity between primers or probes and the surveyed sequence. These methods are facile, highly efficient and can rapidly identify mutations in the genomic sequence probed.

The gold-standard genetic test is qPCR and numerous SARS-CoV-2 assays have been described^3^, targeting multiple mutated loci. However, the bottleneck in reagents and consumables, as well as the need for point of care testing during the pandemic has spurred the development of alternative strategies. Of particular interest has been RT-LAMP^4^ which can rapidly amplify RNA at a single, constant temperature with high specificity and sensitivity. However, achieving the specificity required to discriminate single-point mutations using RT-LAMP is challenging, but the emergence of CRISPR-Cas based detection has the potential to achieve this with isothermal nucleic acid amplification in a one-pot reaction^5^.

The discovery of CRISPR (Clustered Regularly Interspaced Short Palindromic Repeats) adaptive immunity in bacteria and the associated nucleases that cleave foreign RNA/DNA, has led to the development of an increasing number of highly specific genetic tests. CRISPR-Cas-based detection utilises CRISPR associated enzymes (Cas) specific to a target nucleic acid; Cas13 for RNA, and Cas12 and Cas9 for DNA or cDNA (by converting RNA using reverse transcriptase). Many of these techniques have been developed commercially for SARS-CoV-2 detection including SHERLOCK (Specific High-sensitivity Enzymatic Reporter un-LOCKing)^5–7^ and DETECTR (DNA Endonuclease-Targeted CRISPR Trans Reporter)^8^.

Given the threat of VOCs to vaccine efficacy, and their more general threat to at risk populations, any interventions that will help rapidly identify and limit the introduction and spread of such variants, or enable the acceleration of the public health responses, can be an important tool in public health management. Consequently, rapid variant-specific testing is a high priority challenge. Despite this, none have been widely implemented except for the TaqPath qPCR assay, which serendipitously possessed S-gene dropout behaviour with Alpha^9^ and some Omicron variants. This coincidental property of the qPCR assay with these variants has proved to be a valuable proxy to rapidly highlight likely introductions, enabling enhanced testing, contact tracing and isolation and the rapid assessment of case growth. However, this assay was only employed in a sub-section of testing labs in the UK and whilst the S-dropout is otherwise non-specific, its utility in early tracking of these variants clearly highlighted the value of direct variant detection.

Based on the recently developed CRISPR-Cas assay SHERLOCK^6^, here we develop a readily adaptable and rapid molecular diagnostic strategy applicable to existing VOCs, that can be readily employed for newly identified variants. In combination with the power of genomic sequencing, this can aid public health management and allow timely and informed interventions that can help minimise the risk of concerning immune escape variants becoming established.

We thus describe the development of a generalisable approach for variant of concern molecular detection (VarLOCK). We describe optimisation for each mutation of concern through the addition of chemicals, reaction temperature and gRNA target sequence length for unambiguous results, and importantly demonstrate validation and implementation with the Cardiff University COVID19 Testing Service on patient samples. We demonstrate the ability to employ these assays with national wastewater surveillance to identify and map temporal and geographic population-level variant prevalence. We show this approach, when combined with LAMP amplification, could be employed widely for near-patient variant-specific diagnostic testing. The rapid adaptability of VarLOCK for new and emerging VOCs is demonstrated with the development of an Omicron specific assay within 2 weeks of WHO designating it as a VOC. This may contribute to current public health efforts by enabling more rapid identification of Omicron infection, as well as providing a generalisable approach for future VOCs.

## Results

### Concept of the VarLOCK assay

**Figure 1:**
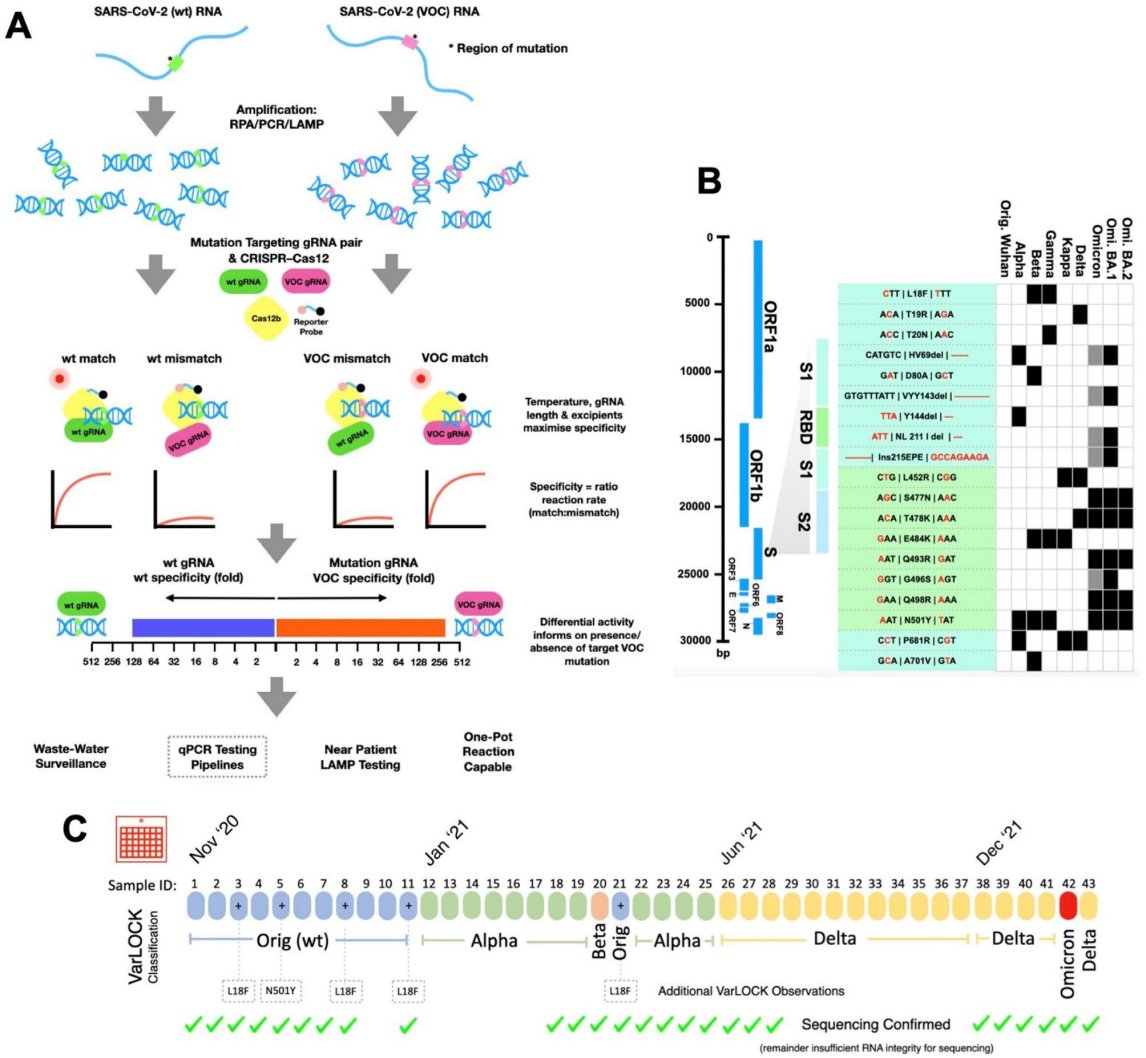
SARS-CoV-2 Variant of Concern identification by VarLOCK assay. (**A**) illustration of the VarLOCK assay. The region with wildtype (green) or mutant (pink) sequence of SARS-CoV-2 RNA is amplified by either RPA, LAMP or PCR. Amplified DNA fragments are subjected to SHERLOCK assay with a pair of gRNAs matching the wildtype sequence (wt gRNA, green) or the mutant sequence (VOC gRNA, pink). We aimed to find conditions in which only a perfect match between the gRNA and the amplified DNA can trigger a collateral nuclease activation of the Cas12b protein to cleave a quenched fluorescent reporter. This allows nuclease activity to be monitored by the increase in fluorescence. The ratiometric response of an unknown SARS-CoV-2 variant sample indicates the presence, or absence, of the targeted mutation and thus enables identification of the VOC. (**B**) Schematic of the genome of SARS-CoV-2 (left) with blow-up of the Spike protein encoding region showing the location of RBD, S1 and S2. Nineteen mutation sites were chosen for VarLOCK assay for which gRNA pairs were designed and optimised. Nucleotide sequences encoding the relevant amino acids are shown in the table and the nucleotide substitutions/deletions are marked in red. The association of the mutations with various variants of concern is indicated in the table, creating a barcode-like identification matrix. (**C**) Application of this approach to saliva samples successfully identifies original Wuhan, Alpha, Beta, Delta and Omicron variants with results confirmed by sequencing.

SARS-CoV-2 variants are characterised by specific sets of mutations selected in the course of virus evolution. Sub-variants diverge from the main VOC clades through acquisition of additional mutations, and as some mutations are not fully penetrant individual samples can have diverged mutational profiles. By identifying the mutations present in the sample’s genome, the variant can be identified. Mutations may be unique and associated with a single VOC, but many other mutations are shared between different VOCs potentially associated with the selective advantage they confer. In particular, variants can be differentiated by the composition of mutations in the spike protein gene (S-gene) (Table 1, Fig. 1B).

**Table 1:** gRNA target sequences surrounding the variant sites.

Variant sites on spike protein	Name of gRNA	Target sequences	Strand	Mutation position to PAM	Number of changes vs wildtype sequence
L18F/T20N/T19R	L18-T20 wt	ttaAT**C**TTA**C**AA**C**CAGAACTCAATTA	Plus		
	L18-T20N	ttaAT**T**TTACAA**A**CAGAACTCAATTA	Plus	3/10	2
	T19R	ttaATCTTA**G**AACCAGAACTCAATTA	Plus	7	1
69/70del	69-70 wt	ttgGTCCCAGAGA**CATGTA**TAGCATG	Minus		
	69-70del	ttgGTCCCAGAGATAGCATG**GAACCA**	Minus	11	6
D80A	D80 wt	ttg**A**TAACCCTGTCCTACCATTTAAT	Plus		
	D80A	ttg**C**TAACCCTGTCCTACCATTTAAT	Plus	1	1
144del/143-145del	144 wt	tttGG**GTGTTTATT**ACCACAAAAACA	Plus		
	144del	tttGGGTGTTTACCACAAAAACA**ACA**	Plus	10	3
	143-145del	tttGGACCACAAAAACA**ACAAAAGTT**	Plus	3	9
N212I/212del/215EPEins	N211 wt	tta**ATT**TAGTGCGTGATCTCCCTCAG	Plus		
	N211I-212del-215EPEins	ttaTAGTGCGTGA**GCCAGAAGA**TCTC	Plus	1	12
L45R	l452 wt	ttaCC**T**GTATAGATTGTTTAGGAAGT	Plus		
	L452R	ttaCC**G**GTATAGATTGTTTAGGAAGT	Plus	3	1
S477N/T478K/T478K	T478 wt	ttaCAAGGT**G**TG**C**TACCGGCCTGATA	Minus		
	S477N-T478K	ttaCAAGGT**T**TG**T**TACCGGCCTGATA	Minus	7/10	2
	T478K	ttaCAAGGT**T**TGCTACCGGCCTGATA	Minus	7	1
E484K	E484 wt	ttaAAACCTT**C**AACACCATTACAAGG	Minus		
	E484K	ttaAAACCTT**T**AACACCATTACAAGG	Minus	8	1
Q493R/G496S	Q493-G496 wt	tttAC**A**ATCATAT**G**GTTTCCAACCCA	Plus		
	Q493R-G496S	tttAC**G**ATCATAT**A**GTTTCCGACCCA	Plus	3/11	2
Q498R-N501Y/N501Y	N501 wt	ttcC**A**ACCCACT**A**ATGGTGTTGGTTA	plus		
	Q498R-N501Y	ttcC**G**ACCCACT**T**ATGGTGTTGGTCA	Plus	2/10	2
	N501Y	ttcCAACCCACT**T**ATGGTGTTGGTTA	Plus	10	1
P681R	P681 wt	ttcTC**C**TCGGCGGGCACGTAGTGTAG	Plus		
	P681R	ttcTC**G**TCGGCGGGCACGTAGTGTAG	Plus	3	1
A701V	A701 wt	ttgGTG**C**AGAAAATTCAGTTCCTTAC	Plus		
	A701V	ttgGTG**T**AGAAAATTCAGTTGCTTAC	Plus	4	1

The PAM sequence is shown lower case. The nucleotides substituted in the variants and their corresponding nucleotides in the original Wuhan SARS-CoV-2 sequence are shown in bold. Both deleted nucleotides and the shifted sequences at 69-70del, 143-145del, 144del and 212del are also shown in bold.

CRISPR cleavage is sequence specific and guided by the targeting sequence of the guide RNA (gRNA), and thus by comparing the cleavage using wildtype and mutant specific guide sequences, we reasoned that the mutational state of the investigated region could be determined. We employed a two-pot assay with prior amplification of the target by PCR, RPA or LAMP in the first pot and AapCas12b mediated detection in the second. VarLOCK detection utilises gRNAs recognizing a specific DNA target sequence, a gRNA-guided Cas12b nuclease and a quenched DNA probe that fluoresces when cleaved by the Cas12b when the correct target sequence is bound and cleaved, leading to increased fluorescence, indicating the presence of target DNA. Anti-contamination procedures were followed for the handling of amplified target DNA to restrict false positive results. We designed primers to amplify the target sequences and gRNAs to specifically match wildtype and mutant sequences to enable the SHERLOCK method to cleave with Cas12b quenched fluorescent probes only when a specific match is present. We designed assays for various S-gene mutations namely L18F/T20N, T19R, 69-70del, D80A, 143-145del, 144del, N211I/212del/215EPEins, L452R, S477N/T478K, T478K, E484K, Q493R/G496S, Q498R/N501Y, N501Y, P681R and A701V to enable VOCs to be detected (Fig. 1C). To account for potential non-specific cleavage and reporting, a second reaction with the wildtype sequence specific gRNA was run in parallel, with the differential response informing on the likely presence or absence of the target mutation.

### Selection of variant-defining mutation sites in the S gene

Mutations of SARS-CoV-2 are acquired during genome replication and occur randomly across the whole genome. The SARS-CoV-2 spike (S) protein plays a crucial role in receptor recognition through the receptor-binding domain (RBD), followed by membrane fusion. Mutations in the spike protein likely account for the increased transmissibility and may lead to decreased protection from immunisation and natural infection^10^. We therefore focused for VarLOCK on the differential mutations located in the S gene.

First, to identify the variant-specific Spike protein mutations in the SARS-CoV-2, we compared the S-gene sequences found in Alpha, Beta, Gamma, Delta and Omicron VOCs and Kappa (VOI-B1.617.1) and selected 19 mutation sites that are VOC characteristic and contain a Cas12b protospacer adjacent motif (PAM) site (TTN) in their vicinity to allow CRISPR-based detection assays (Fig. 1A). We then designed 16 pairs of gRNAs for the 19 selected mutation sites (Table 1). Each gRNA pair targets the same region of the S gene, comprising the sequence present in either the Wuhan strain (wt) or the VOCs specific mutation (mut) (Table 1).

### SHERLOCK offers limited discrimination of single nucleotide variation

**Figure 2:**
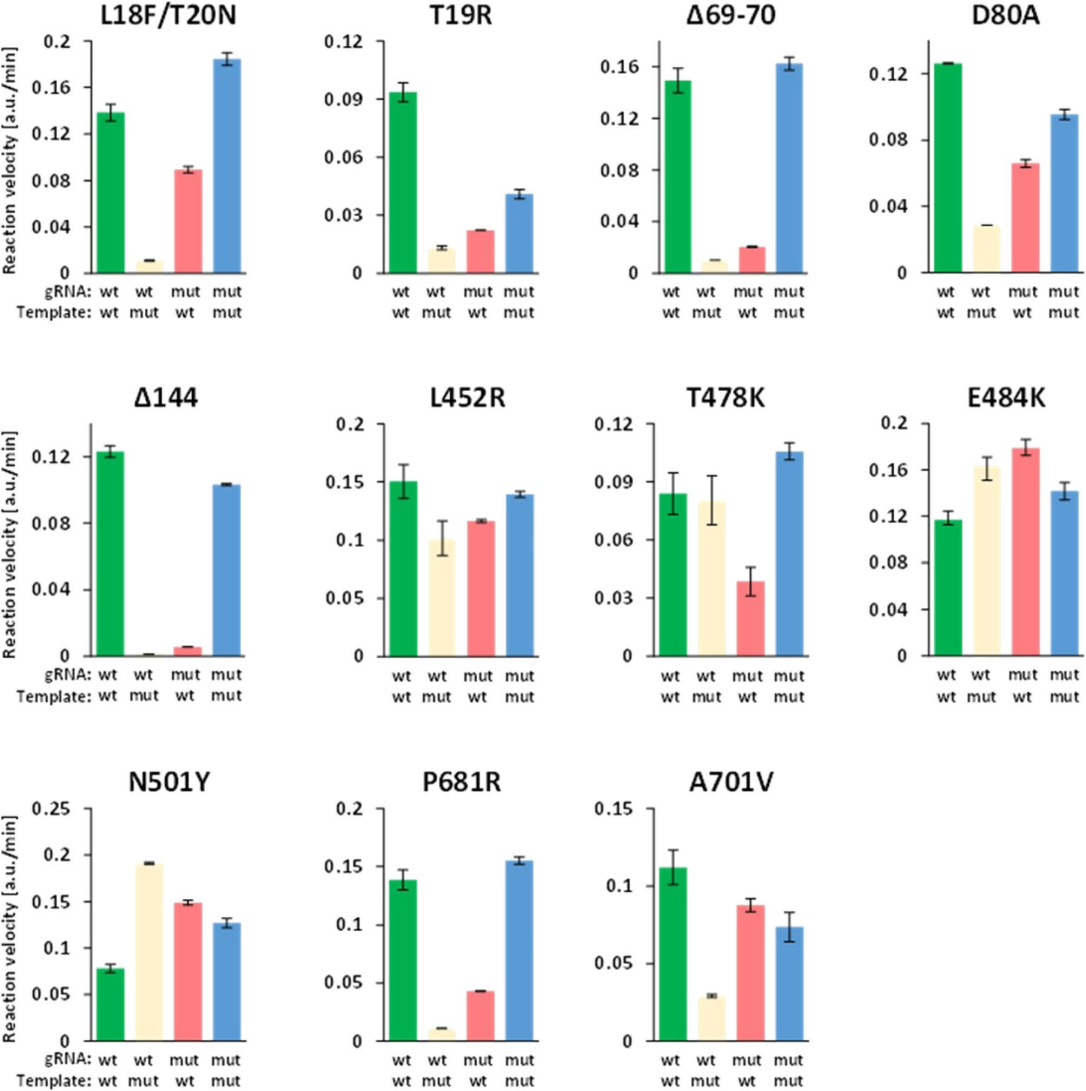
Detection specificity is low for single nucleotide substitution with standard conditions. A representative experiment showing collateral nuclease activities (y axis; arbitrary fluorescence unit increase/min) triggered by wt gRNA matching wt target (wild type detection), wt gRNA matching mutation target (wt cross-reaction), mutation gRNA matching wt target (mutation cross-reaction) and mutation gRNA matching mutation target (mutation detection). Mutations are indicated on the top of each panel. Average and SD are derived from technical duplicates.

First, we tested whether the SHERLOCK assay provides sufficient discrimination for our VarLOCK approach to differentiate specific variant mutations. We performed SHERLOCK detection assays using wildtype (wt) and mutant (mut) specific gRNAs on short synthetic DNA templates containing either wildtype or mutant sequences for the tested SARS-CoV-2 genomic loci, using the reported assay conditions^6^ (Supplementary Table 2 and Fig. 2). For each gRNA pair, four sets of assays were performed (gRNA/template)—wt/wt and mut/mut, to check the on-target performance of the assay, as well as two discordant sets with wt/mut and mut/wt combinations to check for assay crosstalk (specificity). In an ideal case, the assays should provide high reporter activity on matching pairs with only background signal observed for discordant pairs. The detection reactions were followed in real-time by observing an increase in HEX fluorescence coming from the cleaved quenched fluorescent reporter and the reaction velocities were extracted for each of the reactions.

All the assays provided efficient on-target activity, but different targets showed varied levels of discrimination between the discordant pairs. The 69-70del, 144del and P681R assays differentiated the wt and the mutant alleles effectively (>10-fold discrimination). However, other assays offered only limited discrimination (<fourfold) (i.e., D80A or T478K) or failed to discriminate wildtype and mutant templates (i.e., E484K, L452R or N501Y). Of the 8 pairs of gRNAs for the variants containing single nucleotide substitution, only two (T19R and P681R) showed clear discriminative activities between matched and mismatched targets (Fig. 2). Not surprisingly the position of the substitution on the gRNA target sequences relative to PAM and the number of nucleotide exchanges affected the degree of discrimination as observed for the other CRISPR/Cas systems (Table 1). The two deletion sites, 69-70del and 144del, have the greatest power to discriminate the mut from wt. Single nucleotide exchanges could only be efficiently discriminated in the P681R assay, for which the substitution is only 3 bp away from PAM. Interestingly, D80A, for which the substitution is right next to PAM did not show strong discrimination between the wildtype and mutant templates.

### Assay optimisation for efficient discrimination of variants

As observed, the “standard” SHERLOCK conditions afforded only limited discrimination of the VOC-specific mutations. Whereas more extensive genetic changes could be discriminated, single nucleotide exchanges distal to the PAM could not. We therefore surveyed various assay conditions and chemical additives to increase the discriminatory power of the assay. The base-pairing specificity of nucleic acids can be influenced by numerous factors. For example, in PCR reactions, the specificity of primer binding to the template is, amongst other factors, dependent on the annealing temperature used and the reaction ionic strength. We have limited the search to conditions that can be tolerated by the polymerases and enzymes used in the assay to permit a “single pot” reaction.

We selected several common PCR enhancers that are used to alleviate specificity issues in PCR amplification: DMSO, trehalose, Gly-Gly, betaine, 1,2-propanediol, as well as GITC which have been shown to improve amplification efficiency and/or specificity^11–13^. Furthermore, we checked whether specificity could be enhanced by increasing assay temperature or by using gRNAs with a shorter spacer sequence as previously observed for CRISPR/Cas9 specificity^14^.

We selected the gRNAs pairs for L18F/T20N (g18/20 wt and g18/20 mut) and the E484K (g484 wt and g484 mut) as examples for testing various conditions (Supplementary Figs. S1 and S2C). The PCR enhancers including: DMSO, Gly-Gly, betaine (up to 1 M), trehalose, 1,2-propanediol, or a combination of these, did not appear to improve significantly VarLOCK specificity (Supplementary Fig. S1A,B), neither did the NEB Q5 high GC enhancer.

**Figure 3:**
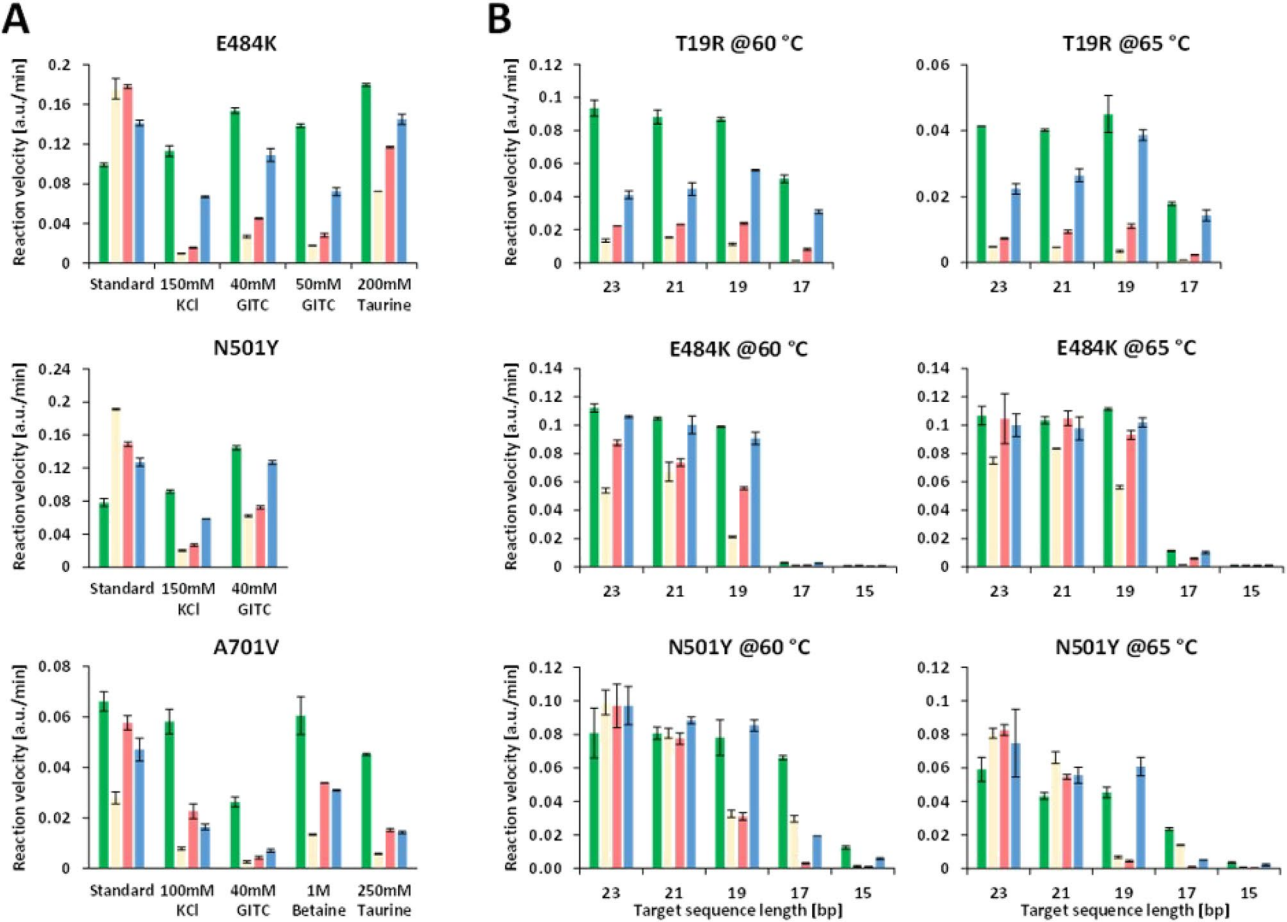
Detection specificity can be improved by interfering stability of intermolecular interaction, gRNA length and reaction temperature. (**A**) Reactions were performed in standard buffer or with the indicated concentrations of additives. Reporter activities triggered by wt or mut gRNA matching or mismatching the targets are calculated as increase of arbitrary fluorescent unit/min. Representative experiments for mutation E484K, N501Y and A701V are shown. Average and SD are derived from technical duplicates. (**B**) reaction performed at 60 $^\circ$C or 65 $^\circ$C. The lengths of target sequences in gRNA are indicated under the x axis. Average and SD are derived from technical duplicates of a representative experiment. Reaction velocity is calculated by using linear regression from the initial reaction curves (signal over reaction time). The reaction velocity measurements for each match and mismatch guide RNA are compared to determine technical specificity of the cleavage reaction.

Strikingly, addition of salts at increasing concentrations improved template discrimination. Increasing salt concentration in the reaction mixture of KCl to 150 mM or supplementing with 40–50 mM GITC significantly improved the specificity for E484K (Fig. 3A and Supplementary Fig. S1B,C). KCl and GITC at a similar concentration also improved N501Y specificity (Fig. 3A and Supplementary Fig. S1D). Surprisingly, both KCl and GITC at similar concentration did not improve the specificity for A701V, but inhibited Cas12b activity altogether (Fig. 3). Taurine at 200 to 250 mM concentration improved specificities moderately for L18F/T20N and E484K (Fig. 3A), but no beneficial effect was observed for A701V (Fig. 3A). Overall, chemical additives only moderately improved VarLOCK specificities for some variant sites but did not show a general effect.

The effect of these salts suggests that the low ionic strength of the buffer may contribute to the lack of specificity, or that unspecific charge interactions between DNA and the ribonucleoprotein (RNP) (Cas12/gRNA) promote stability of the mis-coupled substrate/RNP complex.

### Shorter gRNAs and increased assay temperature improve selectivity

Decreasing the length of the gRNA spacer sequence has previously been shown to improve the specificity of Cas9 cleavage^14^. We tested whether a similar effect for Cas12b could be observed and that shortening the length of the gRNA spacer sequences might increase relative base-paring specificities through reduction of free energies of annealing between gRNA and its target.

We tested assay activity and specificity for 7 variant sites with spacer lengths between 23 and 15 nucleotides in reactions performed at two different temperatures using short double-stranded DNA as mentioned before. At 60 $^\circ$C, activities remained comparable for spacer lengths between 19- and 23-nt for all 7 variant sites tested (Fig. 3B and Supplementary Fig. S2A,C,E,G). Specificities were increased with 19-nt spacers in gRNA for T478K, E484K and N501Y, without affecting the on-target signal strength. Further shortening of the spacers to 17-nt dramatically decreased the activities, except those for L18F/T20N, T19R, L452R and P681R. None of the gRNAs with a spacer length of only 15-nt supported cleavage activity (Fig. 3B and Supplementary Fig. S2A,C,E,G).

Alternatively, increasing temperature could reduce annealing stabilities of mismatched counterparts between gRNA and targets, with minimum effects on matched counterparts. As AapCas12b can tolerate temperatures as high as 100 $^\circ$C^15^, we tried to rerun the reactions using the gRNAs with difference length of spacers at 65 $^\circ$C (Fig. 3B and Supplementary Fig. S2B,D,F,H). In comparison with the reactions at 60 $^\circ$C, increasing assay temperature by 5 $^\circ$C did improve specificities significantly. The most improved variant site was N501Y detected using gRNAs with a 19-nt spacer (Fig. 3B). Saliva and wastewater samples were amplified with the standard RT-LAMP protocol before the VarLOCK reaction (a two-pot assay) unless stated otherwise.

### Combination of approaches

As high specificity detection for variant sites L452R and T478K were not achieved by either altering gRNA length or temperature, we decided to test if KCl, GITC and taurine at concentrations that were optimal for E484K and N501Y could also provide a benefit in combination with gRNA length (Supplementary Fig. S3A and B). For L452R gRNAs with 21-, 19- and 17-nt spacers, both GITC and KCl drastically improved specificities with moderate reduction of activities as a compensation (Supplementary Fig. S3A). Taurine also increased specificities, but to a lesser extent compared to GITC and KCl, however. We calculated the ratio of activities derived from gRNA/Target-matched and mismatched reactions as an indicator of specificities (Supplementary Fig. S3B). We saw that the effects of the chemicals were both on gRNAs that match the wildtype sequence (g452wt) and the variant sequence (g452mut). To evaluate the overall specificities, we took the averages of the two ratios (g452wt and g452mut) as a combined fold difference for comparison across all reaction conditions. Almost 8-fold discrimination was achieved for 21-nt gRNA with 50 mM GITC and 21-nt and 17-nt gRNA with 150 mM KCl (Supplementary Fig. S3B). Similarly, testing on T478K showed that all the three chemicals improved specificities (Supplementary Fig. S3C). In this case, activities remained at similar levels in reactions with GITC, taurine and KCl, compared to the standard reaction, at least for 23- and 21-nt gRNAs (Supplementary Fig. S3C). GITC and KCl reduced the activities slightly for 19-nt gRNAs. The overall fold-differences for matched/mismatched reaction reached 10-fold with 21-nt gRNA with both GITC and KCl (Supplementary Fig. S3D).

**Figure 4:**
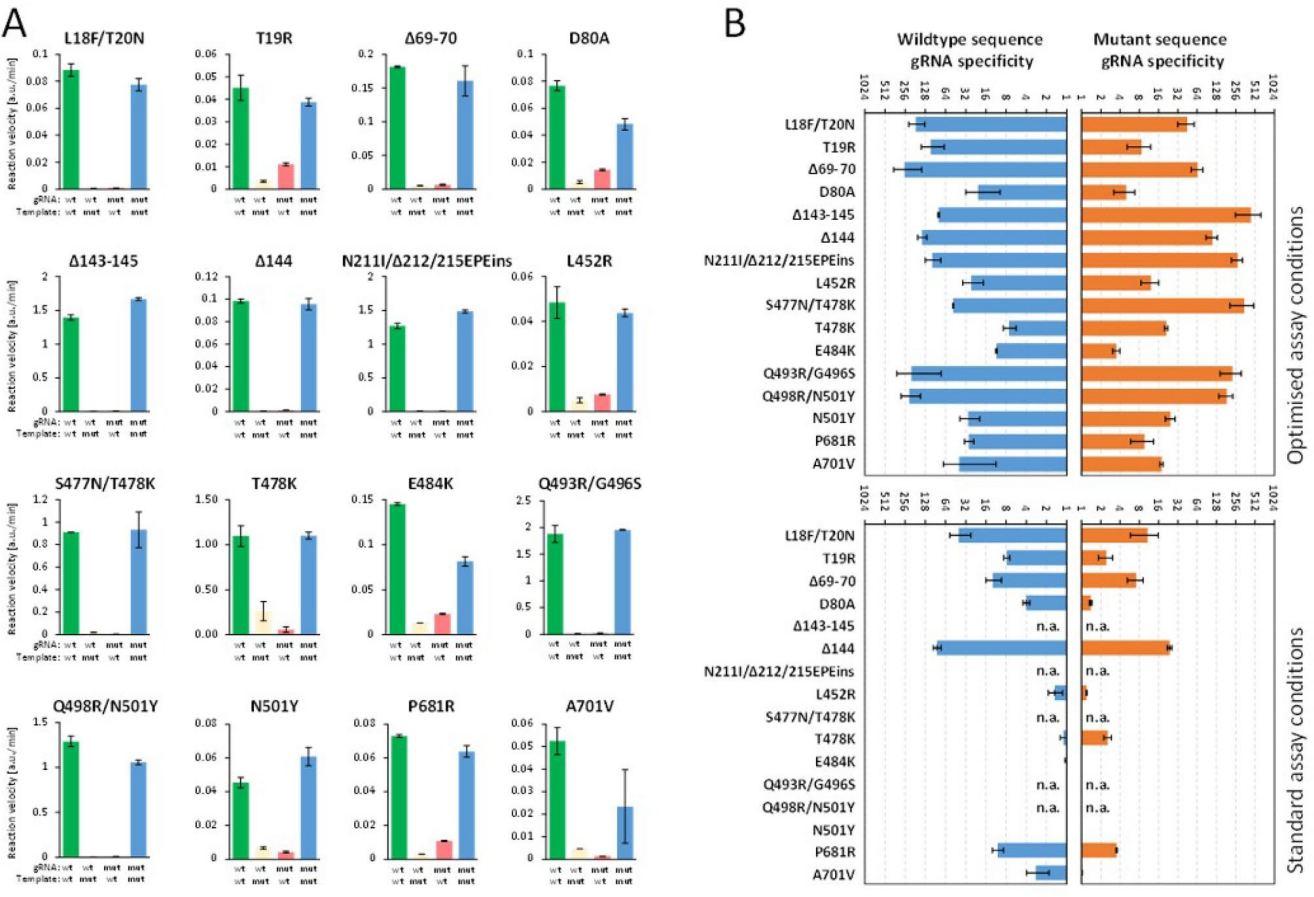
VarLOCK assay sensitively discriminates single nucleotide exchanges. (**A**) Specificity plots for representative examples for each mutation performed under optimised conditions (as listed in Table 2). Average and SD are derived from technical duplicates. (**B**) Specificity factors of the gRNA (wt blue bars and mutation in orange) are presented as ratio of the two reaction activities triggered by matched and mismatched target DNA. Please note that the ratios are displayed on a log scale. The bar graph shows average, and SD of the ratios derived from 2 to 5 biological repeats.

Taken together, for the variant sites with more than two nucleotide changes, L18F/T20N, 69-70del and 144del, specificities can be achieved in standard buffer at 65 $^\circ$C with 23-nt gRNA (see a summary in Fig. 4). Two sites with only single nucleotide substitutions, T19R and P681, can also work well in standard reaction run at 65 $^\circ$C with 23-, 21- and 19-nt gRNAs targeting sequences. L452R, T478K and N501Y require shorter gRNA, chemical additives and higher temperature to achieve specificity with overall fold-difference above 7.

### Adaptation of the VarLOCK with lateral flow assay

After optimisation of the VarLOCK assays with fluorescence detection, which can be easily applied to large scale screening, we asked whether this assay could be adapted to point of care lateral flow assay. We chose two mutation sites, 69-70del, which probes for a 6-nucleotide deletion, and N501Y with just a single nucleotide substitution. As shown, specific detection had been clearly observed for both assays. A very faint non-specific band developed in both no target control (N) and mismatch targets, which may reflect the property of the batch of the dipstick used. Quantitative image analysis of band intensity found equivalent signal in these two scenarios in comparison to the VOC match (Supplementary Fig. S5).

### Validation of the VarLOCK assay using saliva samples

Next, we sought to check the performance of the VarLOCK assay on positive saliva samples identified at Cardiff University’s COVID-19 screening service. We randomly selected 11, 14 and 15 positive samples collected in Nov 2020, between Feb and May 2021, and between Jun and Aug 2021, respectively. Based on the historical data, in the three selected time periods, the Wuhan, Alpha and Delta variants were dominant in the population, respectively. We used the VarLOCK assay to probe the presence of 6 mutations that should provide sufficient information to identify the variant present. Among these, we selected one mutation specific for each variant: 144del for Alpha, A701V for Beta, L18F/T20N for Gamma and T19R for Delta variants. We also included two additional mutations found within the RBD region of the S gene, T478K, which is unique for the Delta variant, and N501Y, which is shared between Alpha, Beta and Gamma variants. These mutations are suggested to be responsible for increased infection rates and reduced protection by vaccination and antibody therapies^16,17^.

**Figure 5:**
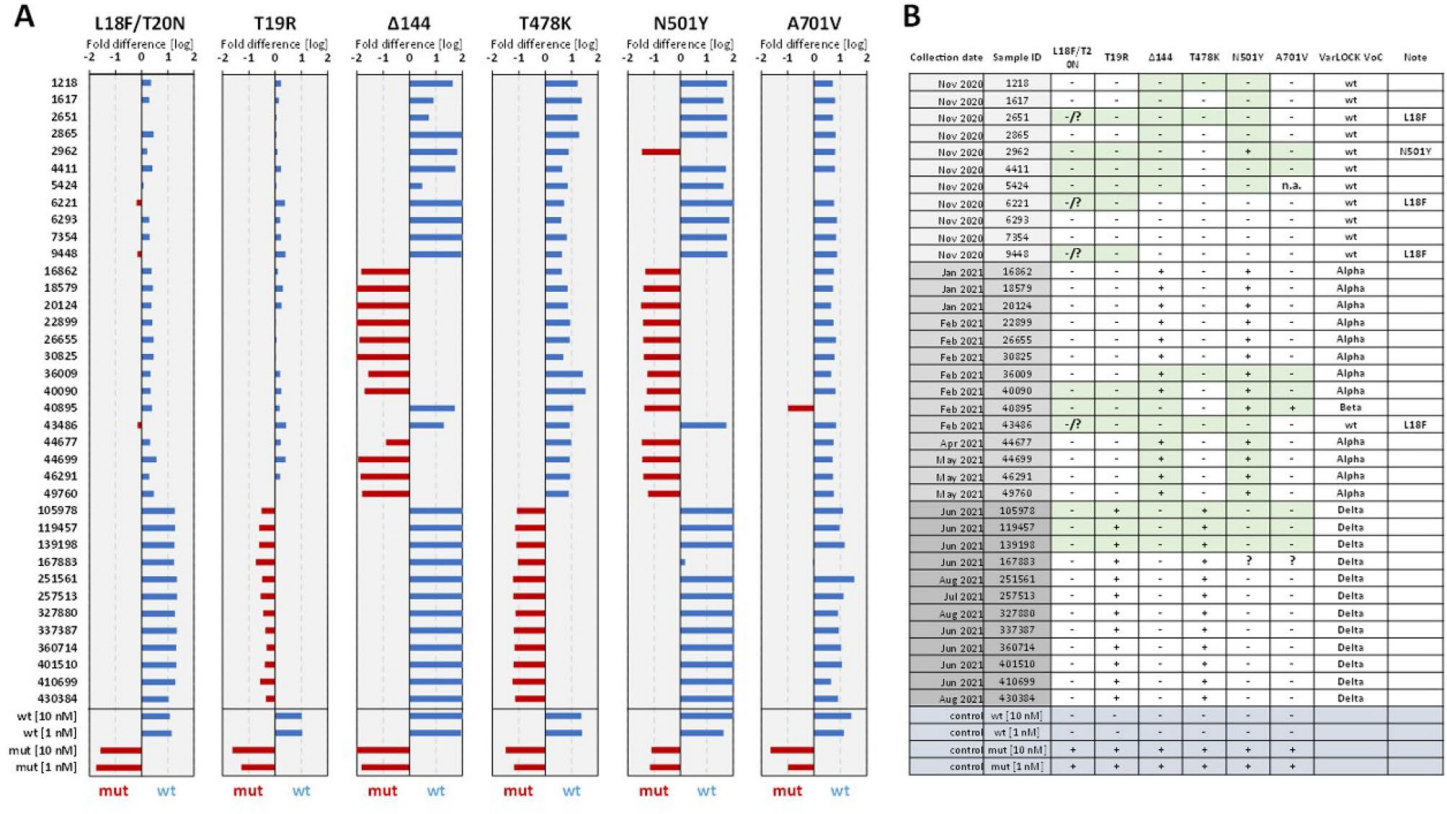
Variant identification of the positive saliva samples. (**A**) VarLOCK assay results showing dominant wildtype signal (blue) or mutant signal (red), calculated as Log_10_ of fold (wt/mut) differences. Samples are grouped in three time periods as indicated with different shading of greyness. (**B**) VarLOCK detection summary for the analysed samples with VOC assignment. Sanger sequencing was used to confirm the variants for samples and amplicons shaded in green.

The relevant regions of interests were amplified by RT-PCR from saliva samples and the products were subjected to VarLOCK assays. The results are presented as Log_10_ of the ratio of the two VarLOCK activities obtained with wt gRNA and mut gRNA. This provides a simple method to visualise samples as wildtype or mutant, based on whether the Log_10_ value is above or below 0 respectively (Fig. 5). This presentation was evaluated with wildtype and mutant control targets, and both 1 and 10 nM control targets showed similar Log_10_ value, indicating a tolerance of variabilities of RT-qPCR amplification in all 6 mutation sites (Fig. 5).

144del, a signature for Alpha variant, showed that 12 out of 14 samples collected between February and May 2021 were assigned as Alpha. These 12 samples also carried the N501Y mutation as typically found in Alpha (Fig. 5). The presence of A701V mutation (Beta) was only detected in one sample (40895). This sample also carried the N501Y mutation. The A701V assay also produced ambiguous results for 167883. To understand the cause of the ambiguity, we sequenced this sample. Sequencing suggested that, although this sample had a small portion of C to T substitution, the sample may contain more than one sequences (Supplementary Fig. S7).

The L18F/T20N assay detecting a Gamma specific mutation, did not indicate the presence of this mutation in any of the samples tested, nor did it conclusively assign the wt sequence in 4 of the tested samples, namely 2651, 6221, 9448 and 43486, suggesting the presence of a different mutation from L18F/T20N. Indeed, Sanger sequencing of these PCR products showed that all these 4 samples carried the spike protein L18F mutation (single nucleotide substitution from C to T) (a representative sequencing result shown in Supplementary Fig. S7B). The Delta variant probe T19R showed that all 12 samples had this mutation (Supplementary Fig. S5). Sanger sequencing of a few selected templates was used to validate the VarLOCK results (Supplementary Fig. S6). Indeed, samples which tested positive for mutation in the VarLOCK assay contained the expected sequences when sequenced (Fig. 5B). Interestingly, although no VOCs were designated before December 2020, we detected 3 samples with the L18F mutation out of the 11 samples collected in November 2020. Twelve of the 14 samples collected between January and May 2021 were Alpha variants and all the 12 samples collected between June and August 2021 were Delta variants.

### VarLOCK wastewater

Wastewater monitoring for the detection of SARS-CoV-2 has become an important tool, complementing hospital and community-based clinical surveillance to identify the presence and relative rates of transmission of COVID-19 infection within the population of a defined geographical area^18^. Wales was one of the first countries to implement nation-wide surveillance of COVID-19 in wastewater, which covered all of Wales’ major urban areas and circa 80% of the Welsh population^19^. Globally, there is increasing interest in local and national wastewater surveillance to monitor the effect of public health interventions and monitor or inform of possible new local outbreaks ahead of, or as an alternative to, mass testing. We hypothesised that the VarLOCK assay could be used to monitor and map the prevalence of variants of concern in close to real time. In principle, this could provide speed, coverage or resource advantages over symptomatic testing and subsequent sequencing of selected positive cases. This could be especially useful where sequencing resources are scarce or when national COVID-19 caseload is high in a highly vaccinated population, but the emergence or introduction of immune-escape variants with known mutations of concern could risk an increase in severe disease. To evaluate the feasibility of applying VarLOCK to identify VOCs in wastewater, VarLOCK assays were used to discriminate between the original Wuhan, Alpha, Beta and Delta variants retrospectively in wastewater samples collected from three wastewater treatment works (WwTWs) serving the cities of Cardiff, Swansea and Newport (all urban centres in South Wales), which were collected weekly from November 2020 to November 2021, as part of the COVID-19 Welsh Wastewater monitoring programme.

Unlike samples isolated from an individual, where the presence of only one SARS-CoV-2 variant is normally expected, wastewater samples contain a mixture of numerous individual samples. In addition, in contrast to samples isolated from an individual, the viral genomes in the wastewater are highly fragmented, and the samples are complex and contain nucleic acids from a wide range of sources, posing a hurdle for sequencing. Weekly samples were processed with the VarLOCK assay to determine the prevalence of different variants. For this the relevant regions were amplified with RT-PCR and the products analysed with assays probing for 69-70del, 144del (Alpha), T478K (Delta) and A701V (Beta).

**Figure 6:**
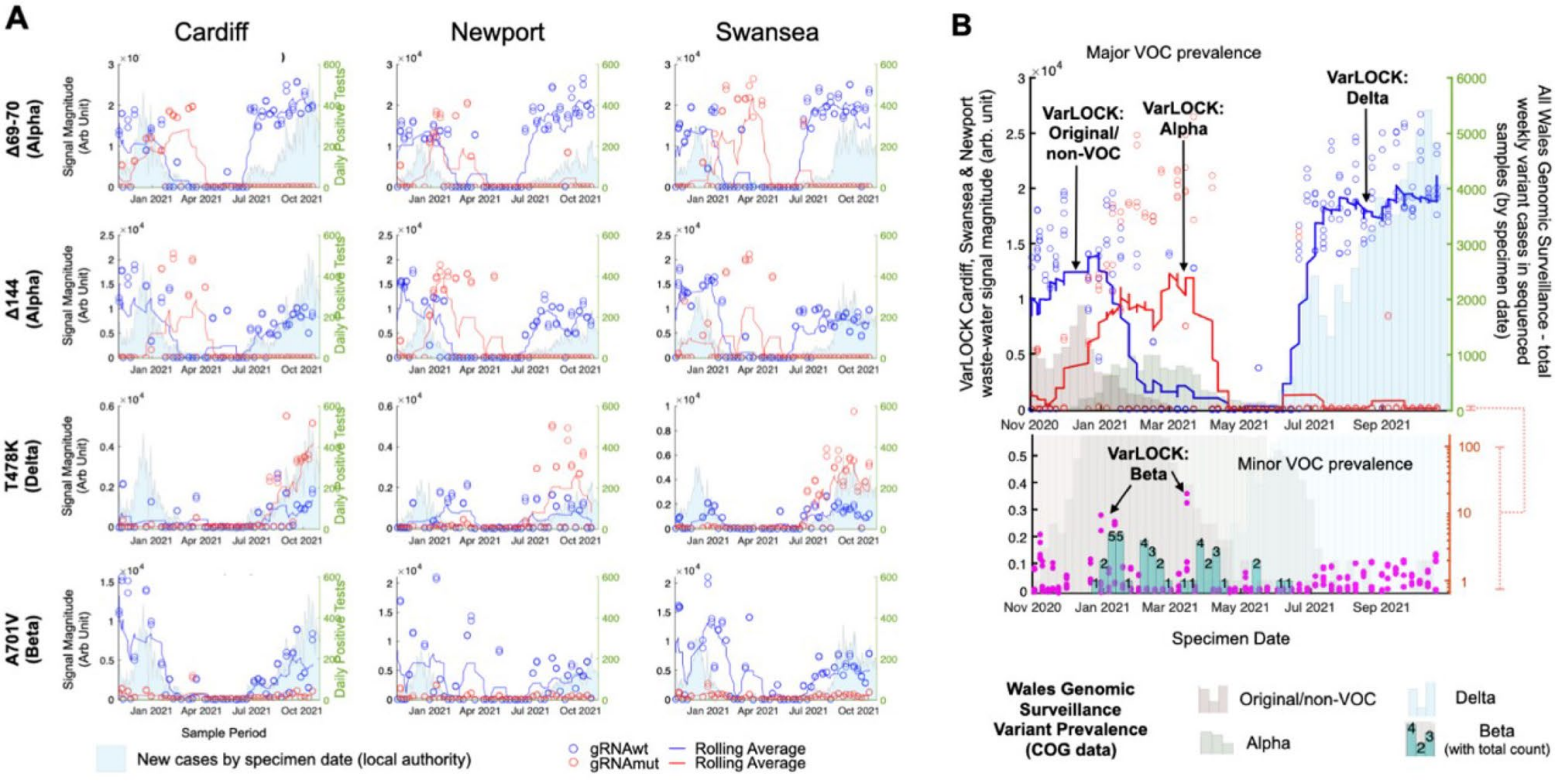
VarLOCK analysis of wastewater samples. Wastewater samples collected weekly between November 2020 and November 2021 at three urban centres in South Wales were analysed with VarLOCK assays for original variant, Alpha, Beta and Delta VOCs. The results showed successful application of VarLOCK for variant monitoring in wastewater with the VarLOCK variant signal reflecting dominant periods of the original variant, Alpha and then Delta VOCs in the country (**B**). The growth of the Alpha variant can be seen to arrive first in Cardiff, followed by Newport and then Swansea (**A**). Against a high background of Alpha variant VarLOCK signal indicative of the presence of the low prevalence Beta variant is observed in the wastewater samples correlating with sequencing identified clusters in Wales (**B**).

For the Cardiff, Newport and Swansea samples, a differential VarLOCK mutation-specific signal is observed with time, reflecting the respective introduction and then dominance of the original variant, Alpha and then Delta VOCs in the country (Fig. 6A). The sequential introduction and growth of the Alpha variant in Cardiff, followed by Newport and then Swansea is notable and likely reflective of the tightening of Government COVID19 restrictions in Wales through December 2020 and January 2021, with limited household mixing, stay-at-home orders and restricted travel accounting for a slow-down in seeding events with reduced inter-city mixing. In contrast Delta VOC arrival and dominance appears more uniformly across the cities with this variant becoming established in the U.K. amidst lighter restrictions. Across the three cities the combined VarLOCK VOC profile maps extremely well to the all- Wales genomic surveillance data catalogued by the COVID-19 Genomics UK Consortium (COG) (Fig. 6B top panel). Positive SARS-CoV-2 detection and VOC identification is also made when community prevalence is low (e.g. June 2021) indicating the sensitivity of the approach.

Precise caseload quantification by waste-water monitoring is known to be challenging^18^. For example, here some wastewater samples recorded no observable viral material despite being collected during a period of known high prevalence. We attributed this to variability of conditions and/or interplay of timing in the wastewater-processing and sample collection and this would require further investigation (these null data points have been retained in the presented data to avoid subjective bias). However, VarLOCK VOC identification in wastewater is observed to be effective at both high and low community caseload throughout the study. Further to this, we observed VarLOCK identification of the presence of mutations characteristic of the Beta variant in wastewater samples. Community transmission of Beta VOC was limited in Wales with the variant not becoming established in the presence of high Alpha caseload and restrictions reducing transmission, with only 40 cases across all of Wales recorded by sequencing during this study period. The VarLOCK Beta VOC response temporally correlates with recorded sequence clusters in Wales (Fig. 6B lower panel). Here, the VarLOCK VOC assay was able to identify the presence of the sparse Beta variant against a backdrop of high Alpha caseload, showing the sensitivity of the approach. Whilst this study was retrospective, collectively these data highlight the promising potential for utilising wastewater VarLOCK monitoring for near-real-time VOC monitoring.

### Detection of Omicron variant and subvariants

A novel SARS-CoV-2 variant B.1.1.529 was identified in Southern Africa and flagged as containing a potentially concerning number of spike mutations on 23rd November 2021 following sequence identification in Botswana (3 genomes), and Hong Kong ex S. Africa (1 genome, partial)^20^. South African public health recorded an extremely large and rapid increase in SARS-CoV-2 cases and test positivity in Gauteng Province for the week of 14–20 November 2021^21^ with this rapidly attributed to Omicron by South Africa Centre for Epidemic Response and Innovation^22,23^. It was designated by WHO and named Omicron VOC on 26th November 2021. Local epidemiology studies showed significant re-infection risk in a population with relatively low vaccination coverage^24^ and early laboratory studies indicated significant reduction in neutralising ability of vaccine and prior-infection induced antibodies^25–27^. This reinfection and incomplete vaccine protection risk has been borne out in populations of higher vaccination coverage following Omicron introduction in the U.K. with the variant rapidly becoming dominant against a background of high Delta caseload. The rapid spread of Omicron across the globe highlights the global public health challenge in containing VOCs following their identification. Whilst sequencing has been indispensable in the scientific effort in monitoring, tracking, understanding and tackling Omicron, the associated lag has hindered containment or implementation of targeted proactive measures, where rapid molecular diagnostics could arguably provide important speed and scale advantages. S-gene target failure (SGTF) has proved a useful proxy for this, enabling rapid assessment of early Omicron case growth in U.K. (2–3 day doubling)^28^ and determining dominance in England by 14th December 2021^29,30^. However, at the time SGTF was limited to four main qPCR laboratories in the U.K. (Alderley Park, Glasgow, Milton Keynes, and Newcastle Lighthouse Laboratories) for community (Pillar 2) testing, so many areas and cases remained under-reported until community transmission is well established. Furthermore STGF occurs due to fortuitous primer design, and cannot be relied upon for identification of future variants.

In this context we applied our generalisable VOC detection strategy to Omicron, developing and implementing an assay able to differentiate Omicron from delta, wt and other variants and additionally positively detect and distinguish between Omicron sub-variants BA.1 and BA.2, where BA.2 is undetectable by SGTF. Within 4 days from receiving the oligonucleotides, we successfully developed 5 VarLOCK assays to probe for the presence of Omicron specific mutations in the S gene: 143-145del (BA.1), N211I/212del/215EPEins (BA.1), S477N/T478K (BA.1 and BA.2), Q493A/G496S (BA.1) and Q498R/N501Y (BA.1 and BA.2) (Tables 1 and 2). First, the assays were tested and optimized with synthetic dsDNA templates (Fig. 4). In addition, given that the same S gene locations are found differently mutated in other variants, we cross-validated the specificity of the Omicron assays against the templates containing other VOC specific sequences (Fig. 4). All the assays showed excellent specificity, ranging between 49.1 and 347.9-fold discrimination.

**Table 2:** A summary of detection specificities in optimised conditions.

Mutations (N)	Variants detected	gRNA set	Target length	Reaction temp. (°C)	Reaction conditions (additives)
L18F(C>T)/T20N(C>A)	Gamma	L18-T20 wt::L18F-T20N	23,**21**,19	65	Standard
T19R(C>G)	Delta	T19 wt::T19R	23,21,**19**	65	Standard
69-70del(CATGTC)	Alpha	69-70 wt::69-70del	23	65	Standard
D80A(A>C)	Beta	D80 wt::D90A	23	65	Standard
143-145del(GTGTTTATT)	Omicron	144 wt::143-145del	23	65	Standard
144del(TTA)	Alpha	144 wt::144del	23	65	Standard
N211I-212del-215EPEins	Omicron	N211 wt::N211I-212del-215EPEins	23	65	Standard
L452R(T>G)	Delta	L452 wt::L452R	**21**,19	65	**50 mM GITC**, 150 mM KCl
S477N(G>A)/T478K(C>A)	Omicron	T478 wt::S477-8mut	21	65	**150 mM KCl**
T478K(C>A)	Delta	T478 wt::T478K	**21**,19	65	**150 mM KCl**, 50 mM GITC
E484K(G>A)	Beta Gamma	E484 wt::E484K	23	60	**150 mM KCl**, 50 mM GITC
Q493R/G496S	Omicron	Q493-G496 wt::Q493R-G496S	21	65	Standard
Q498R(G>A)/N501Y(A>T)	Omicron	N501 wt::Q498R-N501Y	23	65	Standard
N501Y(A>T)	Alpha Beta Gamma	N501 wt::N501Y	19	65	Standard
P681R(C>G)	Delta	P681 wt::P681R	**23**,21,19	65	Standard
A701V(C>T)	Beta	A701 wt::A701V	23	65	Standard

The table shows positions and amino acid substitutions in the spike protein and the corresponding nucleotide mutations (in brackets). The VOC, the gRNA sets, the target lengths in gRNA (the preferred length are shown in Bold). Reaction temperature (in Celsius) and additives (preferred additives in Bold) are indicated in the table.

**Figure 7:**
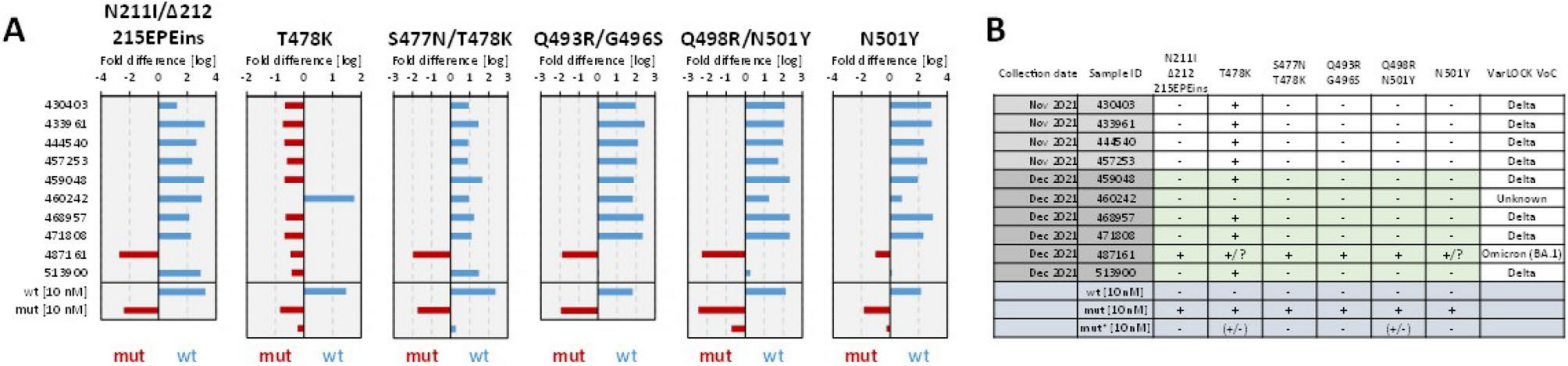
VarLOCK identification of Omicron variant in saliva samples. (**A**) VarLOCK assay results showing dominant wildtype signal (blue) or mutant signal (red), calculated as Log_10_ of fold (wt/mut) differences. For the regions where mutations are present in other VOCs, an additional control was used with alternative template for the other mutants (mut*) (**B**) VarLOCK detection summary for the analysed samples with VOC assignment. Illumina sequencing was used to confirm the variants for samples shaded in green.

Subsequently, we applied the VarLOCK Omicron detection to 10 positive saliva samples collected between the last week of November and mid December 2021. In one of the samples—487161, the VarLOCK assays identified the presence of N211I/212del/215EPEins, S477N/T478K, Q493A/G496S, Q498R/N501Y Omicron specific mutations with good confidence. The 6 most recent positive samples were selected for whole-genome SARS-CoV-2 sequencing using the NimaGen protocol. In the VarLOCK-identified Omicron saliva sample, deep sequencing reconfirmed the presence of the detected mutations and Nextstrain server assigned the sample as Omicron (21K). The other 5 sequenced samples were assigned to the Delta clade (21J), thus reconfirming VarLOCK VOC detection results obtained (Fig. 7).

## Discussion

As pandemics such as the COVID-19 pandemic develop, causative viruses such as SARS-CoV-2 continue to mutate, and novel variants arise. Further variants with transmission advantages afforded by immune escape could therefore emerge. Here we show that once mutational sequences are known, VarLOCK provides a readily implementable means to monitor their introduction and progression. This can provide new tools in the management of the pandemics, such as variant specific testing for travel or events; or enabling more rapid implementation of enhanced public health measures, such as surge testing or enhanced contact tracing upon early knowledge of importation or community transmission of VOCs. In this context, whilst genomic sequencing affords unparalleled insight into viral identification and lineage tracking, especially in the evolution of novel variants, it requires relatively high viral loads and is subject to delay and capacity limits, even in well-resourced nations. This lag may contribute to variants gaining an unstoppable foothold diminishing the effectiveness of public health containment efforts, as seen in the U.K. with Delta and Omicron VOCs where spread has been rapid. Consequently, faster as well as more time and resource efficient variant identification methods can contribute to more rapid and targeted public health responses when necessary.

In this regard, the development of new variant specific VarLOCK assays is rapid. The Omicron detection assays were developed within four days from the receipt of the required oligonucleotides, which can be extremely rapidly designed following identification of concerning genomic sequences. This may also be considered on the basis of predicted mutations that may be of concern to monitor for their emergence. For example, S477N, Q498R, N501Y mutations, as found in Omicron, were predicted to substantially increase ACE2 binding if to arise in combination^31^. Here, the reported Omicron VarLOCK assay was designed and tested with synthetic samples before a positive Omicron case had reached our 3000 sample/day capacity SARS-CoV-2 testing pipeline.

We show here that VarLOCK is applicable to a range of analytical samples, not only enabling variant identification in saliva samples from positive individuals as part of a PCR testing workflow, but that it is also applicable to the monitoring of variant prevalence in pooled population samples. Further, the possibility to use the VarLOCK approach with different amplification approaches (RPA, LAMP and PCR) with fluorescent or colorimetric readout means it can be integrated into PCR testing pipelines, coupled with near patient LAMP testing and is even compatible with lateral flow readout. However, we observed a very faint background band at the ‘test’ line which could not be removed by altering the amount of indicator (Supplementary Fig. S9) or reporter (Supplementary Fig. S10). Further optimisation, alternative source of lateral flow dipsticks and validation would be required for the method.

We have demonstrated that the developed VarLOCK approach is scalable, easily adaptable for different mutations and readily implemented on different sample types including highly fragmented pooled population samples. The VarLOCK approach is able to discriminate single nucleotide exchanges in the identification of VOCs. To achieve this level of specificity optimisation of assay conditions considering gRNA length, reaction temperature and excipients favouring binding discrimination are used to enable enhanced SNP identification. This approach significantly improves the selectivity of previously reported SHERLOCK assays, and by developing targeted VarLOCK assays with a combination of mutation specific gRNAs we are able to identify the mutational pattern present and enable successful VOC identification in infected individuals and in wastewater samples from urban populations. In this regard even low prevalence Beta VOC signatures are observed in South Wales wastewater during early 2021 amidst a background of very high prevalence Alpha infections, highlighting the detection sensitivity of the approach to new variant introductions. The risk of carryover contamination in the two-pot assay was controlled through strict anti-contamination procedures, however this could be enhanced by utilising Uracil DNA Glycosylase (UDG) and dUTPs in the initial target amplification.

The optimised VarLOCK assay is able to discriminate single nucleotide polymorphism in both DNA and RNA species (Supplemtary Fig. S8). Here, we apply it to identify SARS-CoV-2 variants. We show the approach can be rapidly repurposed for new VOCs with the development and implementation of Omicron specific tests, including differentiation of BA.1 and BA.2 sub-types. VarLOCK may also be employed for sensitive and specific identification of other pathogens, for example influenza (A/B), RSV or identification of antibiotic resistance bacteria, wherever genetic identification, or especially SNP or variant discrimination, is of importance. This may contribute to future pandemic preparedness, especially as the approach is readily integrated with existing testing infrastructure. The ability to apply to population-level wastewater samples may also afford the opportunity for multi-pathogen monitoring in communities for enhanced public health surveillance.

In summary, the VarLOCK approach allows rapid implementation and development in response to new pathogens or genetic variants, with the assay being suitable for implementation at point-of-care, for integration into qPCR testing pipelines and application with wastewater surveillance. When combined with pathogen genomic sequencing VarLOCK approaches can rapidly translate sequenced variant information into rapid, sensitive, and specific nucleic acid diagnostics for enhanced public health benefit.

## Methods

### Purification of AapCas12b

pAG001 His6-TwinStrep-SUMO-AapCas12b was a gift from Omar Abudayyeh and Jonathan Gootenberg (Addgene plasmid no. 153162)^5^. The purification procedure of AapCas12b was adapted from the protocol described for TwinStrep-SUMO-LwaCas13a with modifications^32^. Briefly, protein expression was induced by adding 0.5 mM IPTG to transformed BL21-CodonPlus (DE3)-RIL cells (Agilent) and incubated at 18 °C for overnight. The cell pellet from 1 L culture was resuspended in 40 ml Lysis Buffer (20 mM Tris pH7.5, 0.5 M NaCl, 10% Glycerol, 1 mM DTT, and 1 mM PMSF) and sonicated for 10 min at a temperature below 7 °C. Cleared supernatant was incubated with 1 ml Ni-NTA Agarose (Genaxxon Bioscience, S5377) for 1 h at 4 $^\circ$C. After incubation, the slurry was loaded on a gravity flow purification column. The column was washed with 100 ml of Wash Buffer I (20 mM Tris-HCl pH 7.5, 0.5 M NaCl and 10 mM imidazole) followed by 100 ml Wash Buffer II (20 mM Tris-HCl pH 7.5, 0.5 M NaCl and 20 mM Imidazole). The His6-TwinStrep-SUMO-AapCas12b protein was cleaved by His6-Ulp1 (in-house) in SUMO Cleavage Buffer (20 mM Tris pH 7.5, 0.5 M NaCl, 1 mM DTT and 0.15% Igepal CA-630) at 4 °C overnight. Cleaved AapCas12b was eluted in SUMO Cleavage Buffer and dialysed against Dialysis Buffer I (10 mM Tris-HCl pH 7.5, 0.2 M NaCl, 1 mM DTT and 10% glycerol) for 2 h and Dialysis Buffer II (10 mM Tris-HCl pH 7.5, 0.1 M NaCl, 1 mM DTT and 50% glycerol) for 3 h. Protein purity and concentration were estimated with SDS-PAGE.

### gRNA design, synthesis and purification

As the AacCas12b scaffold-based sgRNA has been reported to produce more robust and specific nuclease activity in combination with AapCas12b compared to previously used gRNAs, we designed our gRNA with the AacCas12b gRNA scaffold^5^. In order to synthesise gRNA by in vitro transcription, we placed the T7 promoter sequence at the 5′ end of the gRNA scaffold to form a 115 nt-long oligo (gRNA-sca-temp, see Supplementary Table 1). We then designed 23-nt target sequences using an online program by selecting TTN as PAM sequence (http://www.rgenome.net/cas-designer/)^33^. The target sequences were extended at their 5′ end by adding a 19-nt sequence CAAATCTGAGAAGTGGCAC identical to the 3′ end of the gRNA scaffold. The reverse-compliment oligos of the extended target sequences (Table 1) were used together with a forward T7 promoter oligo (Table 1) and gRNA-sca-temp to generate double-stranded DNA by PCR reaction (with Q5 DNA polymerase, NEB, M0491L). gRNA was synthesised using the PCR product as template with T7 RNA polymerase (Thermo Scientific, EP0111) and template DNA was removed by DNaseI (Thermo Scientific, EN0521) digestion. The gRNAs were further purified using 12% denaturing polyacrylamide gel.

### VarLOCK assay

Inspired by the potential for performing LAMP-SHERLOCK-coupled one-pot assay, we chose to perform the VarLOCK assay in Bst2.0 based buffer omitting dNTP and LAMP primers. For fluorescent assay, each 20 μl VarLOCK reaction contained 20 mM Tris-HCl pH 8.8 at 25 °C, 10 mM (NH_4_)_2_SO_4_, 50 mM KCL, 8 mM MgSO_4_, 0.1% Tween 20, 37.5 nM AapCas12b, 12.5 nM gRNA, 200 nM fluorescent reporter and 10 nM double-stranded target DNA, unless stated otherwise. Fluorescent VarLOCK reaction were either performed at 60 or 65 °C (as indicated) and the FAM fluorescence was recorded every minute for 60 min with LightCycler 96 System (Roche) or ABI QuantStudio 7 (Thermo Fisher Scientific). For comparison, the initial reaction rates are calculated using linear regression in MS Excel by fitting the fluorescence increase during the first 6 min. of the reaction. For lateral flow assay (LFA), all components were the same as above, except 375 nM AapCas12b, 125 nM gRNA, 125 nM LFA reporter and 100 nM double-stranded target DNA were used. LFA samples were incubated at 60 or 65 °C for 60 min and subsequently applied to dipsticks for result readout (Milenia HybriDetect 1, TwistDx, MILENIA01) according to manufacturer’s instruction. Both fluorescent Reporter (5′-HEX-TTTTTTT-3′-IABkFQ/) and LFA reporter (5′-6-FAM-TTTTTTT-3′Bio) were purchased from IDT4 (REF:4)^5^. To optimise the reaction, double-stranded short DNA, annealed using two complimentary oligos, were initially used as targets (sequence listed in Supplementary Table 2). For viral samples, RT-PCR products were generated using primers (listed in Supplementary Table 3) and used as targets in the VarLOCK assay.

### VarLOCK assay with PCR or LAMP products

Synthetic DNA templates harbouring the wt or mutant sequences for PCR or LAMP amplification were synthesised by IDT or by Twist Bioscience. Inactivated wt and mutant SARS-CoV-19 viral particles were provided by Dr. Richard Stanton and the viral RNA extracted with BOMB protocol^34^. LAMP primers were designed using the NEB LAMP primer design tool https://lamp.neb.com/ or Primer Explorer v5.0 https://primerexplorer.jp/e/ (Eiken, Japan). qPCR primers were designed with IDT PrimerQuest Tool. Both LAMP and PCR primers were synthesised by IDT or Merck (listed in Supplementary Table S3). Luna Universal One-Step RT-qPCR Kit (NEB E3005) was used for qPCR. When using viral RNA as template, 1x Luna WarmStart^®^ RT Enzyme Mix was included and a 10 min incubation step at 50 °C was added before PCR cycles. For the LAMP reaction to produce amplicons for VarLOCK, the reaction mix contained 1X isothermal buffer (NEB), 10 mM DTT, 0.4 mg/ml PVP, 60 mM KCl, 5 mM each dNTP, 0.8 M betaine, 0.5 μM SYTO9, 0.32 U/μl Bst v2.0 Warm Start polymerase (NEB), 0.8 μM FIP/BIP, 0.4 μM LF/LB and 0.2 μM F3/B3 primers per 10 μl volume. Artificial templates were used for wild type, Alpha and Beta variants. LAMP amplification was observed on the green channel (490 nm) at 60 °C using a RotorGene thermocycler (Qiagen) for 40 min.

### VarLOCK assay with saliva samples

Saliva samples were extracted in the Cardiff University COVID-19 test laboratory using automated BOMB protocol^34^. A pilot DNA quantification survey showed that typical concentration of the amplicons after RT-qPCR from saliva samples reached the range between 10 and 30 ng/μl, or a sub-picomole/microlitre in molar concentration, which is comfortably within the VarLOCK sensitivity range (1 to 100 fmole/μl). Subsequently we did not quantify DNA concentration of the PCR products and directly used 2 μl for VarLOCK assay in 20 μl reaction. Optimised conditions were used for each of the mutation sites (Fig. 5A).

### Collection and preparation of wastewater samples for VarLOCK assays

Untreated wastewater samples (crude influent) were collected between November 2020 and November 2021 from Dŵr Cymru/Welsh Water WwTWs at Cardiff, Newport and Swansea, as part of the Wales COVID-19 wastewater surveillance programme. On each sampling occasion, 500–1000 ml of crude influent was collected by manual grab sampling between 08.00 and 10.00 h, reflecting peak sewage flow and aiming to capture the highest community faecal load. Suspended solids were then removed from a 150 ml aliquot of each sample by centrifugation (3000*g*, 4 °C, 30 min), and viral nucleic acids collected by subsequent PEG (polyethylene glycol 8000) precipitation for >18 h based on methods described previously^35,36^] with some minor modifications^37^. PEG precipitates collected by centrifugation (10,000*g*, 4 °C, 30 min), and pellets, resuspended in 200 μl of phosphate buffer saline, prior to RNA extraction in the Cardiff University COVID-19 Testing Service laboratory using automated BOMB protocol^34^.

### Automated liquid handling

The QPCR and reaction setup was automated on the Opentrons OT-2 robots. The OT-2 programs for assay assembly are available upon request.

### Sanger and Illumina sequencing

PCR products were sequenced using one of the two PCR primers by Eurofins Genomics sequencing service. Whole genome sequencing was performed utilising NGS library preparation by RT-PCR using the Nimagen protocol, essentially as described (https://www.protocols.io/view/wastewater-sequencing-using-the-easyseq-rc-pcr-sar-bx6dpra6). Subsequently, the libraries were reisolated with SPRI beads, quality of the libraries were checked with TapeStation and the libraries were quantified. Sequencing was performed on Illumina MiSeq 2x250 Nano cartridge.

### Statistics

Each experiment was performed with technical duplicates. Experiments with optimised condition were repeated between 2 and 5 times and Standard Deviation calculated from these biological replicates is presented in Fig. 5B. Tests of the saliva samples were run with technical duplicates.

### Ethical approval

All methods were carried out with all relevant ethical guidelines followed, and any necessary research ethics committee approvals have been obtained. The Ethical approval is obtained from Research Ethics Committee Health and Care Research Wales (IRAS Project ID: 214760). Saliva sample collection, processing and SARS-CoV-2 testing at Cardiff University is approved by ethical approval 18/WA/0089 (Cardiff University BioBank). All necessary participant consent has been obtained and the appropriate institutional forms have been archived. All participants were provided the written informed consent.

### Supplementary Information

Supplementary Information.

## Data Availability

All biological materials used in this work can be obtained from Cardiff University Biobank on request. Data are available on reasonable request from the corresponding authors.
