# Supplementary material for: VarLOCK: sequencing-independent, rapid detection of SARS-CoV-2 variants of concern for point-of-care testing, qPCR pipelines and national wastewater surveillance
Source: Sci Rep. 2023 Nov 27;13:20832. doi: 10.1038/s41598-023-47289-0 (PMC10681975; doi:10.1038/s41598-023-47289-0)
Supplement: Supplementary file 1 — Supplementary Information. [file 41598_2023_47289_MOESM1_ESM.docx]

**VarLOCK - sequencing independent, rapid detection of SARS-CoV-2 variants of concern for point-of-care testing, qPCR pipelines and national wastewater surveillance**

**Supplementary information**

**Authors list**

Xinsheng Nan^1^, Patrick Hardinge^1*^, Sven Hoehn^1^, Shrinivas Nivrutti Dighe^1^, John Ukeri^2^, Darius F Pease^3^, Joshua Griffin^3^, Jessica I Warrington^1,5^, Zack Saud^6^, Emma Hottinger^3^, Gordon Webster^1^, Davey Jones^4^, Peter Kille^1,3^, Andrew Weightman^1^, Richard Stanton^6^, Oliver K Castell^2^, James A.H. Murray^1^, Tomasz P Jurkowski^1,3*^

**Affiliation**

^1^Cardiff School of Biosciences, Cardiff University, Sir Martin Evans Building, Museum Avenue, Cardiff, CF10 3AX, UK.

^2^Cardiff School of Pharmacy and Pharmaceutical Sciences, Cardiff University, Redwood Building, King Edward VII Avenue, Cardiff, CF10 3NB, UK.

^3^COVID-19 screening service, Cardiff University, Sir Martin Evans Building, Museum Avenue, Cardiff, CF10 3AX, UK.

^4^School of Natural Sciences, Bangor University, Bangor, Gwynedd, LL57 2UW, UK.

^5^Current address: Biodexa Pharmaceuticals (Wales) Ltd, 1 Caspian Point, Caspian Way, Cardiff, CF10 4DQ, UK.

^6^ Infection & Immunity, School of Medicine, Cardiff University, Heath Park, Cardiff CF14 4XN, UK

*corresponding authors: [jurkowski@cardiff.ac.uk](mailto:jurkowski@cardiff.ac.uk), [hardingep@cardiff.ac.uk](mailto:hardingep@cardiff.ac.uk)

**Supplementary materials**

**Figures**

Supplementary Figure S1. Effects of chemical additives on detection sensitivities and specificities. Supplementary Figure S2. VarLOCK specificity at different temperatures.

Supplementary Figure S3. VarLOCK reaction performed at 65°C with gRNA containing different lengths of target sequences in the presence of chemical additives.

Supplementary Figure S4. VarLOCK detection with PCR and LAMP products.

Supplementary Figure S5. Adaption of VarLOCK with lateral flow assay.

Supplementary Figure S6. Identification of the VOC with saliva samples collected at three periods of time.

Supplementary Figure S7. Sanger sequencing of selected mutations.

Supplementary Figure S8. Specificity and Technical Accuracy of VarLOCK

Supplementary Figure S9. Reducing the amount of indicator does not solve the background problem.

Supplementary Figure S10. Reducing the amount of reporter does not solve the background problem

**Tables**

Supplementary Table S1. Sequences of guide RNAs.

Supplementary Table S2. Sequences of short target oligonucleotides.

Supplementary Table S3. Sequences of PCR and LAMP oligonucleotides.

Supplementary Table S4. Sequences of DNA synthetic templates for wild type and variants.

Supplementary Table S5. Chemical additives tested for reaction optimisation.

***Supplementary Figure S1. Effects of chemical additives on detection sensitivities and specificities.*** *Activities are shown as the rate of increase of arbitrary fluoresce (units/min).* ***A****, L18F/T20N site.* ***B*** *and* ***C****, E484K site.* ***D****, N501Y site. Wild type detection (wt>wt), wild type cross-reaction (wt>mut), mutation cross-reaction (mut>wt) and mutation detection (mut>mut).*


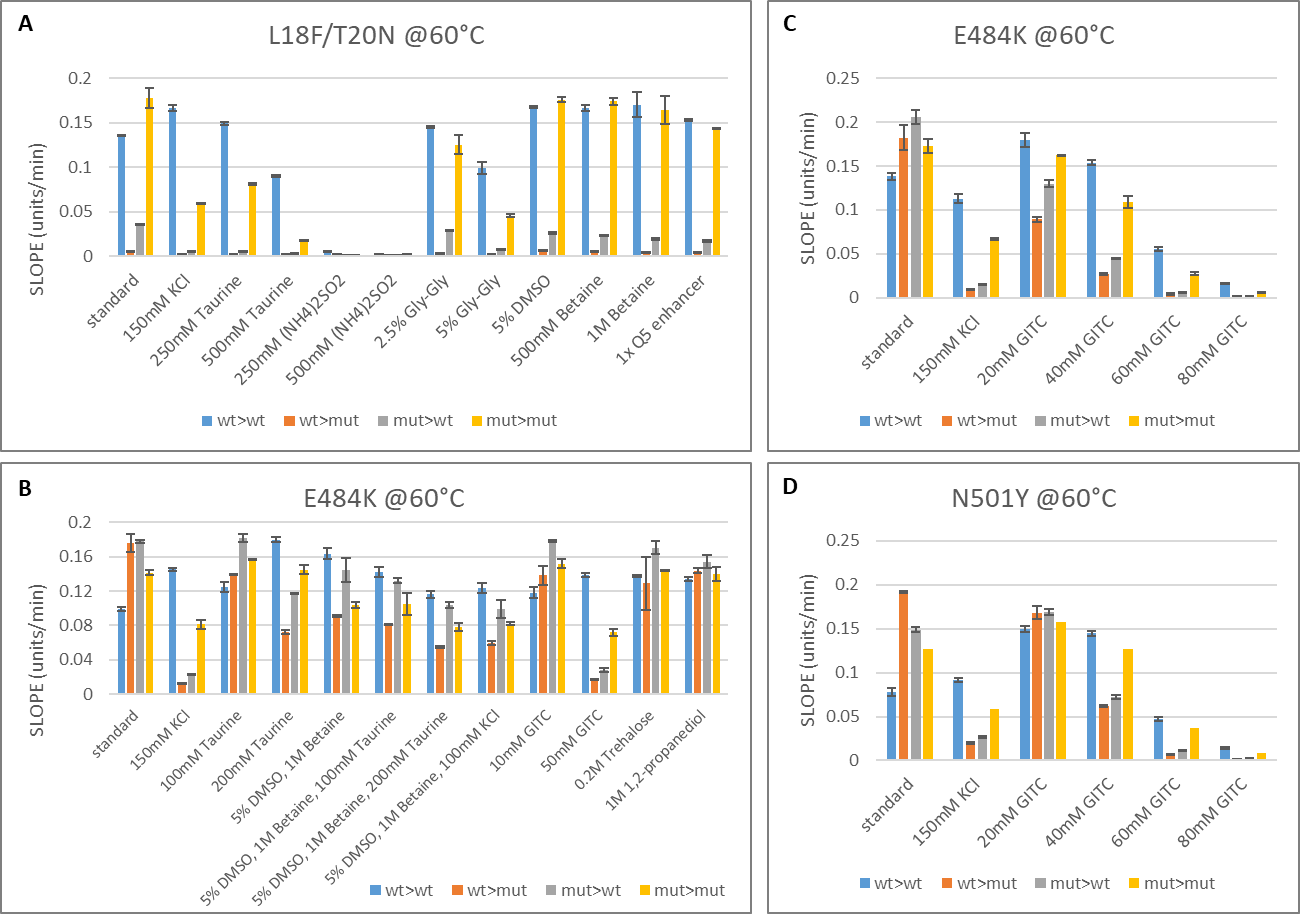


***Supplementary Figure S2. VarLOCK specificity at different temperatures.*** *VarLOCK reaction performed at 60°C (****A****,* ***C****,* ***E****, and* ***G****) and 65°C (****B****,* ***D****,* ***F*** *and* ***H****) with gRNA containing different lengths of target sequences. Wild type detection (wt>wt), wild type cross-reaction (wt>mut), mutation cross-reaction (mut>wt) and mutation detection (mut>mut).*


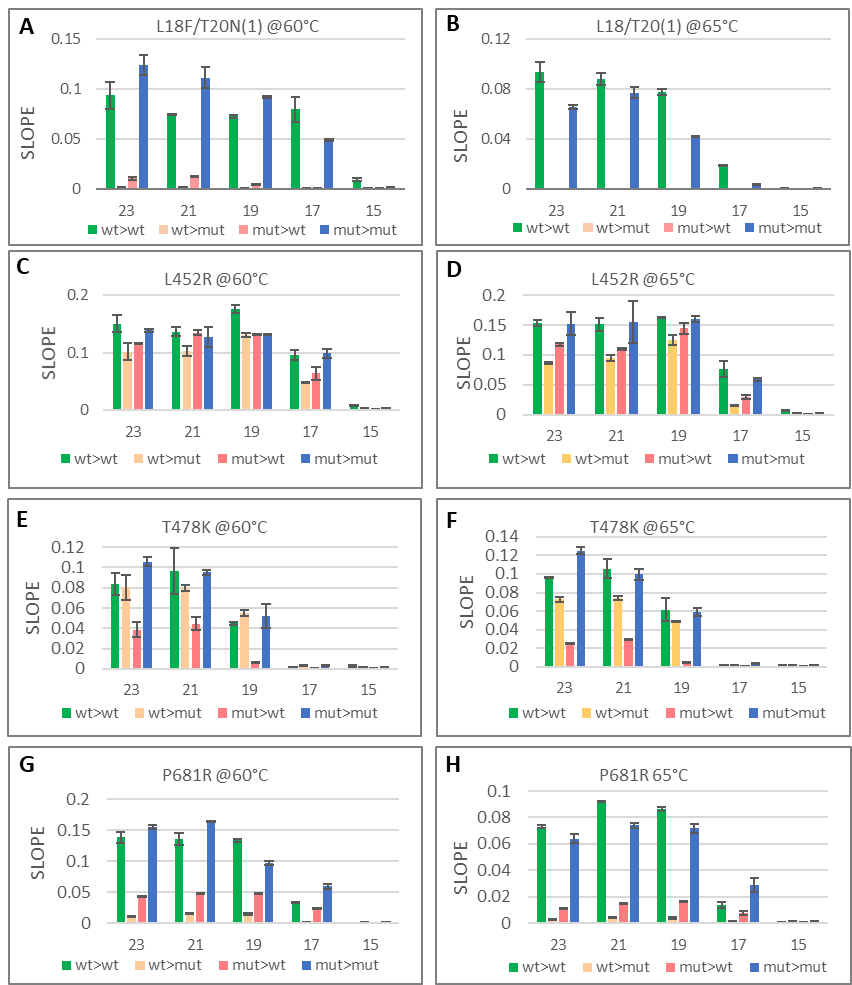


***Supplementary Figure S3. VarLOCK reaction performed at 65°C with gRNA containing different lengths of target sequences in the presence of chemical additives.*** *VarLOCK activities are plotted in panel* ***A*** *and* ***C****. Ratios of VarLOCK activities triggered by gRNA with matched target against mismatched target are shown in panel* ***B*** *and* ***D****. Wild type detection (wt>wt), wild type cross-reaction (wt>mut), mutation cross-reaction (mut>wt) and mutation detection (mut>mut).*


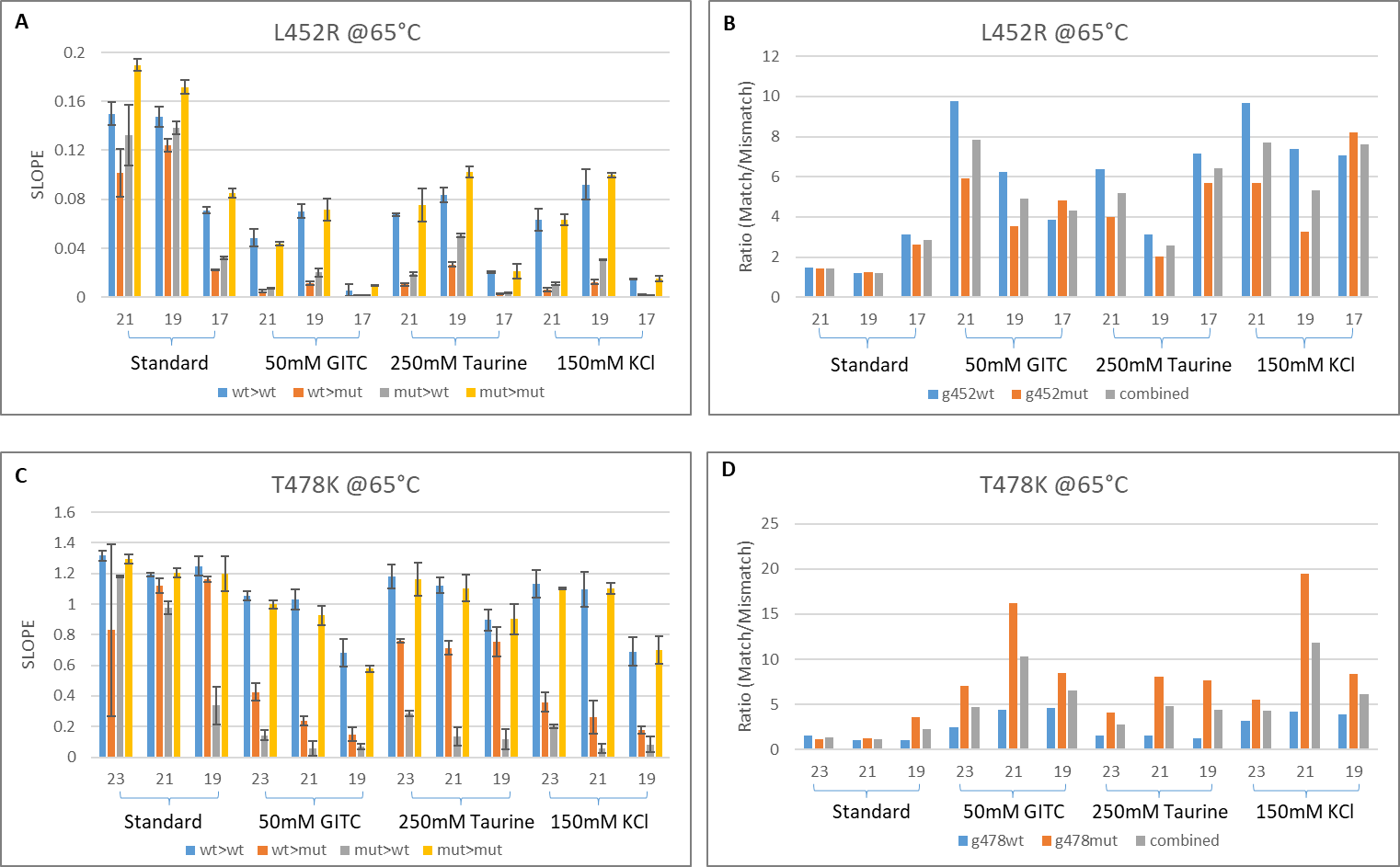


***Supplementary Figure S4. VarLOCK detection with PCR and LAMP products.*** ***A-D****, with PCR products as target for HV69/70del (****A****,****B****) and N501Y (****C****,****D****).* ***E-G****, with LAMP products as targets for HV69/70del (****E****), E484K (****F****) and N501Y (****G****).*
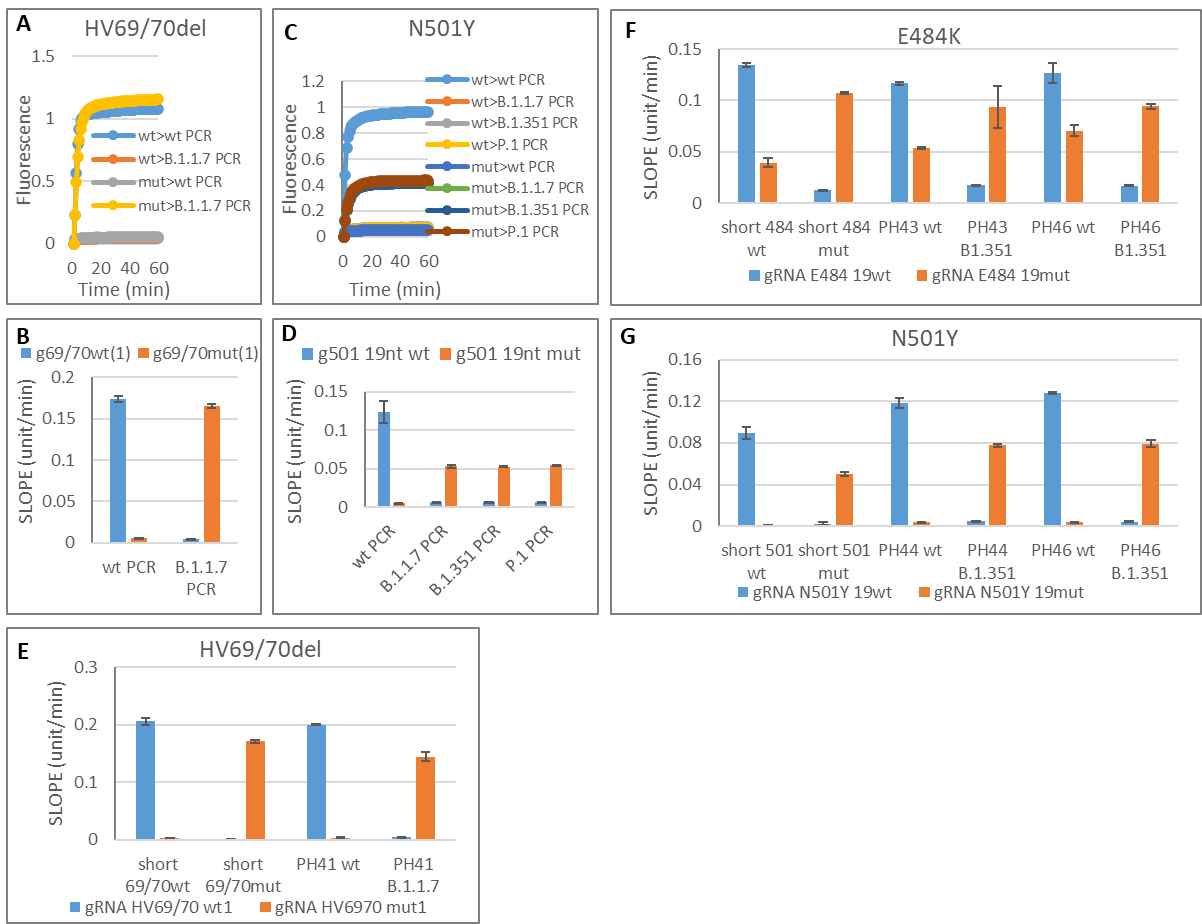


***Supplementary Figure S5. Adaptation of VarLOCK with lateral flow assay****. gRNAs specific to wildtype or mutant sequence were incubated with wildtype (wt), mutant target (mut) or no target (N) in optimised condition (same as Fig 5C and I, except FAM-Biotin reporter was used instead of HEX-IABkFQ reporter). Endpoint reaction was applied to Milenia HybriDetect 1 Dipstick to visualise reactivity. Band intensity measured using ImageJ for relative comparison.*


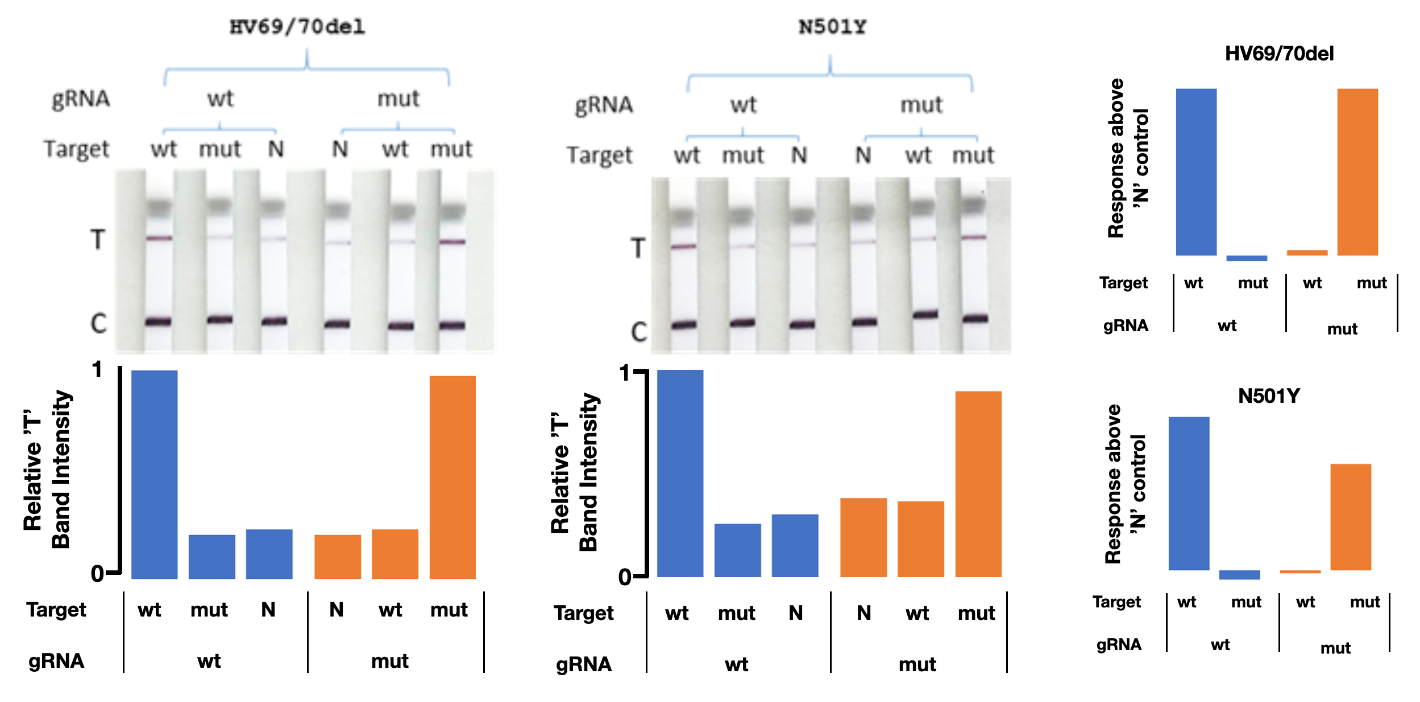


***Supplementary Figure S6. Identification of the VOC with saliva samples collected at three periods of time.*** *Firstly, the prior appearance of Alpha strain, the period when Alpha was in dominance and finally a period when Delta strain was in dominant.*

***Supplementary Figure S7. Sanger sequencing of selected mutations.*** *Sanger sequencing revealed that sample 167883 may contain a mixture of mutations that made the reading unrecognisable in some stretches, or that quality of the sample may be poor for sequencing, compared with a wildtype control sample 119457. A small proportion of A701V (C to T) mutation appeared to be present, indicated with vertical orange line. The affected codon is marked with a box.*

***Supplementary Figure S8. Specificity and Technical Accuracy of VarLOCK.*** *Decreasing amounts of matching short double-stranded oligo targets were used to test the sensitivity of gRNA N501Y in the VarLOCK assay (Panel A). The fluorescence signal ratio between time 0 (background) and time 20 minutes above 5 for the lowest target concentration (Panel B). Therefore, the VarLOCK assay sensitivity was set above 5 for signal ratio. Of the 222 reactions tested from 37 samples, only one reaction failed the sensitivity test (Fig 5 and Panel C) and a further six reactions showed an ambiguous result from the WT and Mut detections (Panel C). Four of these were sequenced and the results indicated new sequence mutations which could explain the unclear variant calling by VarLOCK.*


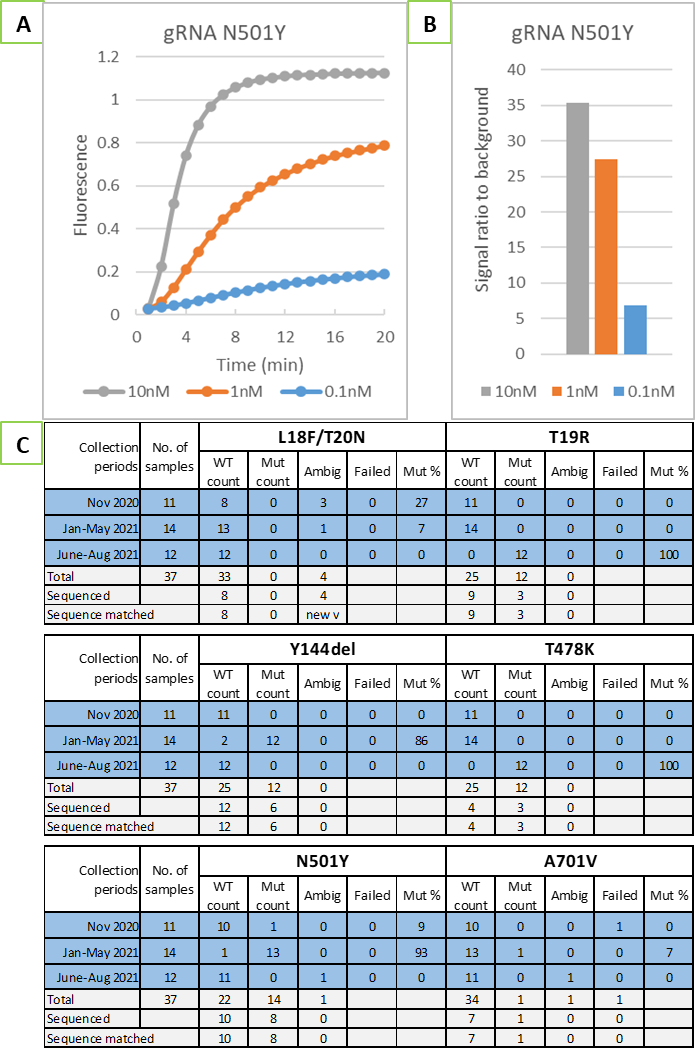


***Supplementary Figure S9. Reducing the amount of indicator does not solve the background problem.*** The high background could be caused by too much indicator in the dipsticks. If this is the case, reducing the amount of indicator to an optimal dosage should reduce the background to a negligible level (Panel **A** shows a hypothetical illustration of LFA affected by the amount of indicator used). *Reducing the amount of the indicator by cutting off part of the strips (up to 20% remaining indicator) resulted in a reduction of band intensity on both T and C proportionately, indicating that background could not be improved by optimising the amount of indicator used (Panel* ***B****).*


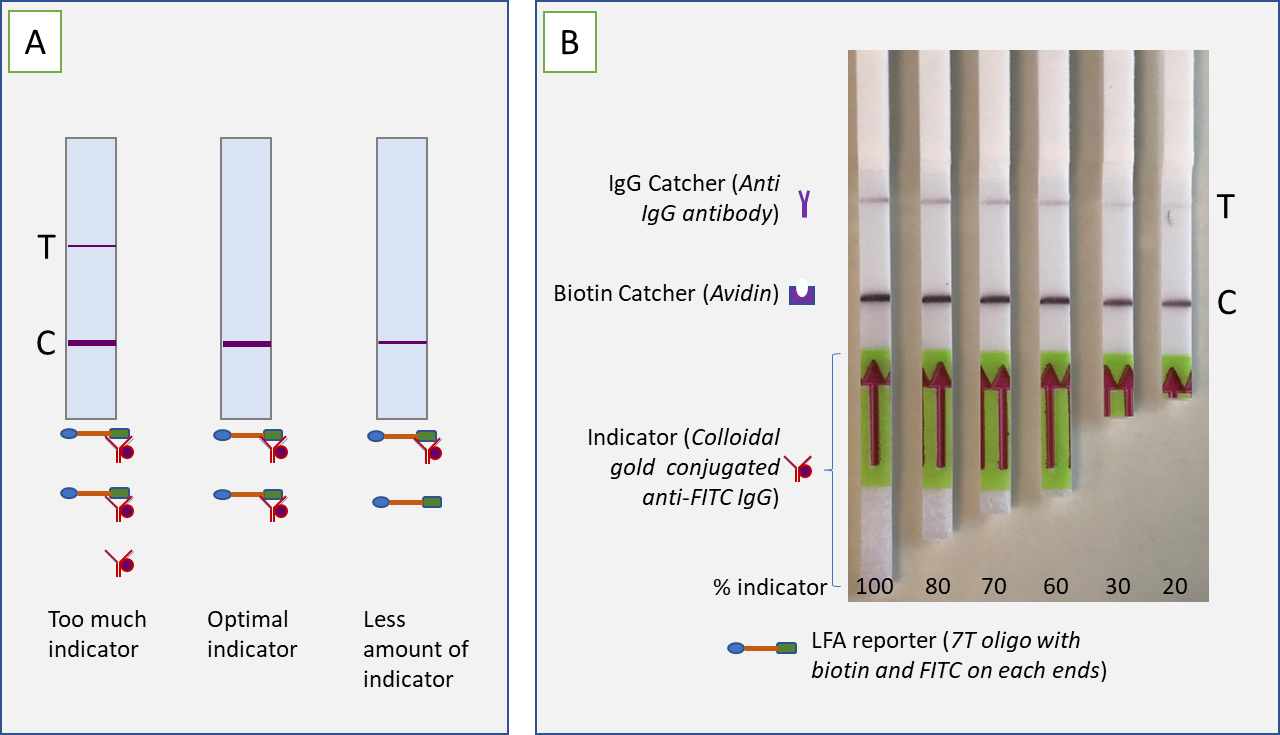


***Supplementary Figure S10. Reducing the amount of reporter does not solve the background problem.*** Without cleavage of the reporter, if excess reporter is used, there is not enough Biotin Catcher (Avidin) to retain the indicator to the C band, which will result in the indicator overflowing to the T band and a false positive. By contrast, if insufficient reporter is used, the indicator also cannot be captured to the C band, which again is captured at the T band (Panel **A** shows a hypothetical illustration). Therefore, the amount of reporter must be optimised. To test this hypothesis in the absence of target template, we used different amounts of reporter in the lateral flow assay. Decreasing the reporter did not reduce the background. Furthermore, if the reporter is further decreased, the colloidal gold conjugated anti-FITC antibody cannot retain at the C band and is captured at the T band (Panel **B**).


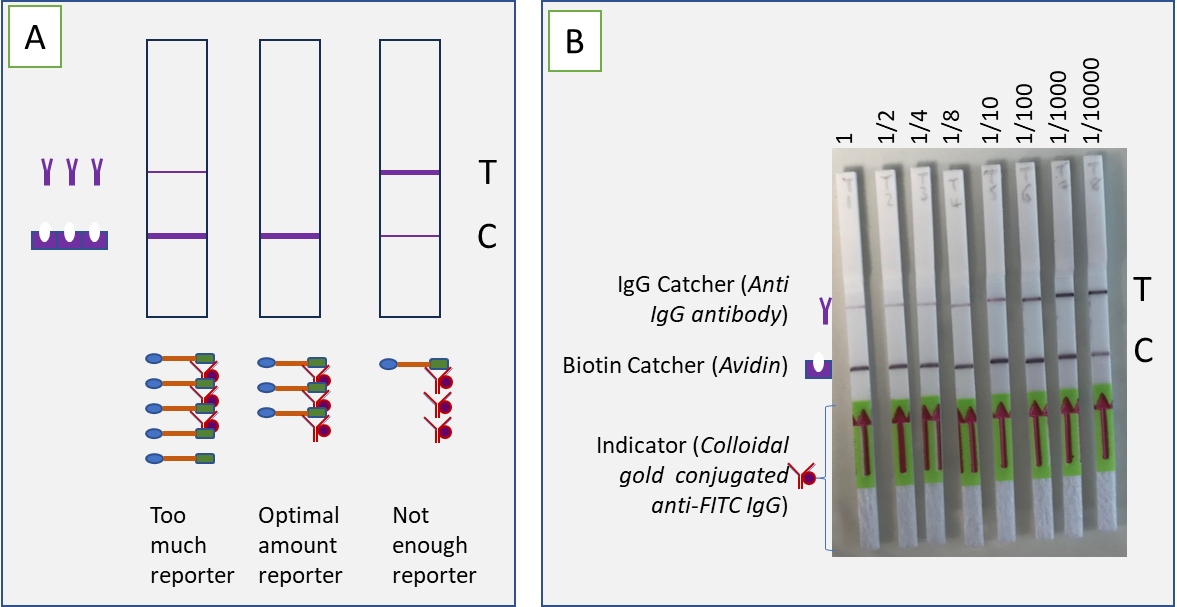


***Supplementary Table S1. Sequences of guide RNAs.*** *Nucleotides highlighted in red indicate the positions of substitutions and deletions.*

***Supplementary Table S2. Sequences of short target oligonucleotides.*** *Nucleotides highlighted in red indicate the positions of substitutions and deletions.*

***Supplementary Table S3. Sequences of PCR and LAMP oligonucleotides.***


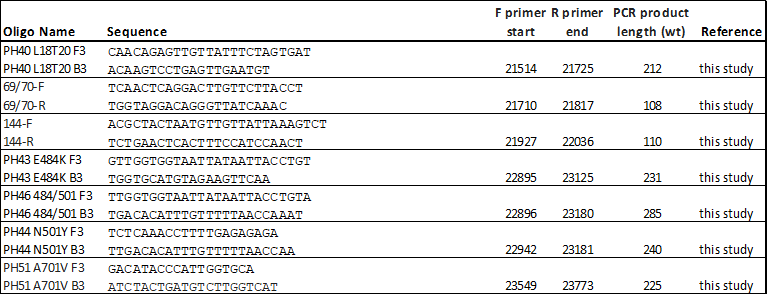

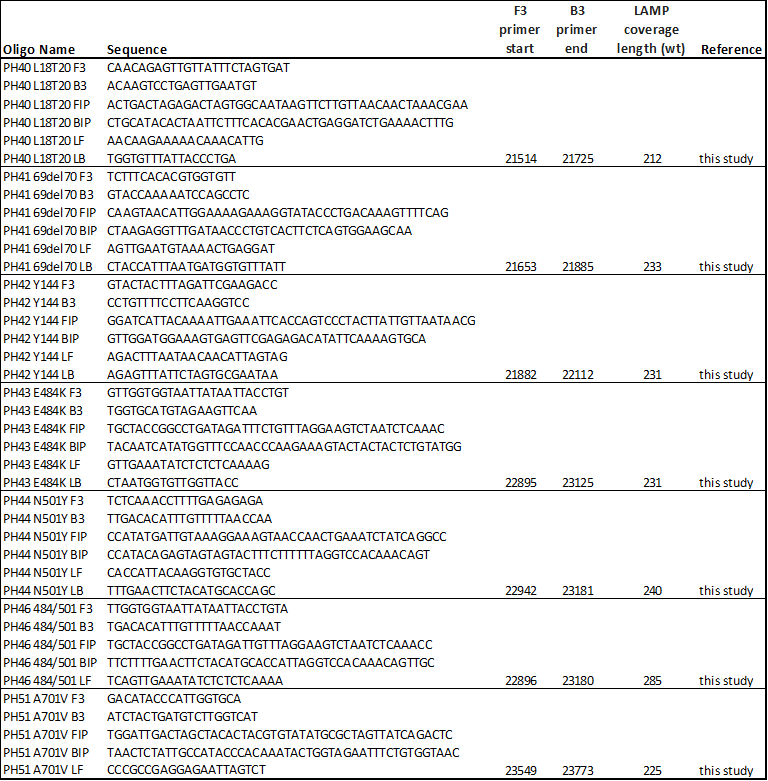


***Supplementary Table S4. Sequences of DNA synthetic templates for wild type and variants.*** *Nucleotides highlighted in red indicate the positions of substitutions and deletions.*


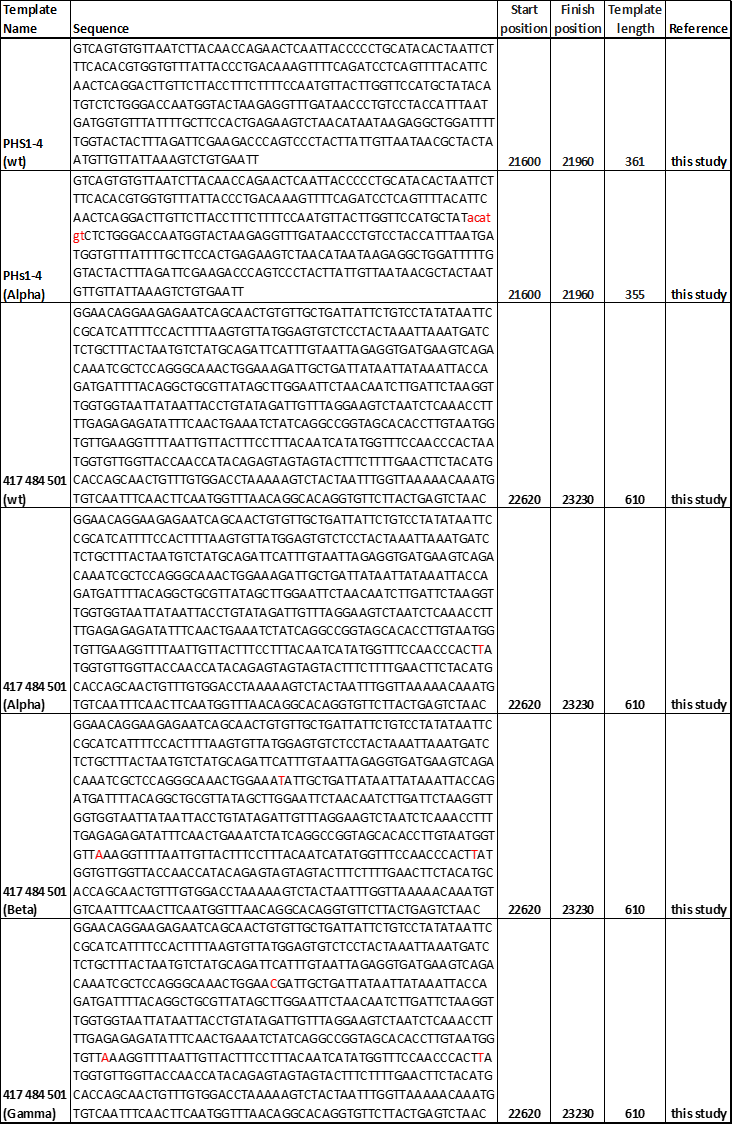


***Supplementary Table S5. Chemical additives tested for reaction optimisation.***
